# Decomposing heterogeneity in disease progression speeds and pathways

**DOI:** 10.1038/s41746-026-02665-8

**Published:** 2026-05-12

**Authors:** Yuichiro Yada, Honda Naoki

**Affiliations:** 1 Laboratory for Data-driven Biology, Nagoya University Graduate School of Medicine, 65, Tsurumai-cho, Showa-ku, Nagoya, Aichi 466-8550 Japan; 2 Institute for Advanced Research, Nagoya Univeristy, Furo-cho, Chikusa-ku, Nagoya, Aichi 464-8601 Japan; 3 Laboratory of Data-driven Biology, Graduate School of Integrated Sciences for Life, Hiroshima University, 1-3-1, Kagamiyama , Higashi-Hiroshima, Hiroshima 739-8526 Japan; 4 Center for One Medicine Innovative Translational Research (COMIT), Nagoya University, 65, Tsurumai-cho, Showa-ku, Nagoya, Aichi 466-8550 Japan

**Keywords:** Computational biology and bioinformatics, Diseases, Neurology, Neuroscience

## Abstract

Understanding why patients with the same diagnosis exhibit markedly different disease progression—some rapidly, others slowly, with distinct symptom patterns—remains a major challenge in medicine. Here, we developed a machine learning framework called DiSPAH (Disease-progression Speed and Pathway Analysis based on a Hidden Markov model) to estimate both the pathway and speed of disease progression in individual patients. DiSPAH models disease progression as continuous-time transitions among latent disease states with a patient-specific progression speed. We applied DiSPAH to longitudinal clinical scores from an amyotrophic lateral sclerosis (ALS) cohort and inferred each patient’s trajectory of the latent disease states and progression speed. These dynamics were associated with baseline clinical features and enabled prediction of future course from first-visit data. Our results highlight that jointly modeling progression pathway and speed improves prediction of heterogeneous disease courses, offering a powerful tool for personalized care and research in ALS and other chronic conditions.

## Introduction

Why do patients with the same diagnosis follow drastically different courses—some deteriorating rapidly, others slowly, and often through entirely different patterns of symptom progression? Understanding and predicting the individual course of disease progression remains one of the most challenging problems in medicine^1–9^. In chronic and currently incurable diseases such as amyotrophic lateral sclerosis (ALS), this variability is not only clinically frustrating but also impedes effective treatment planning, clinical trial design, and patient counseling^8,10,11^. Marked inter-individual heterogeneity in disease course is a pervasive feature of chronic diseases and a key motivation for precision-medicine approaches^12–16^.

**Fig. 1:**
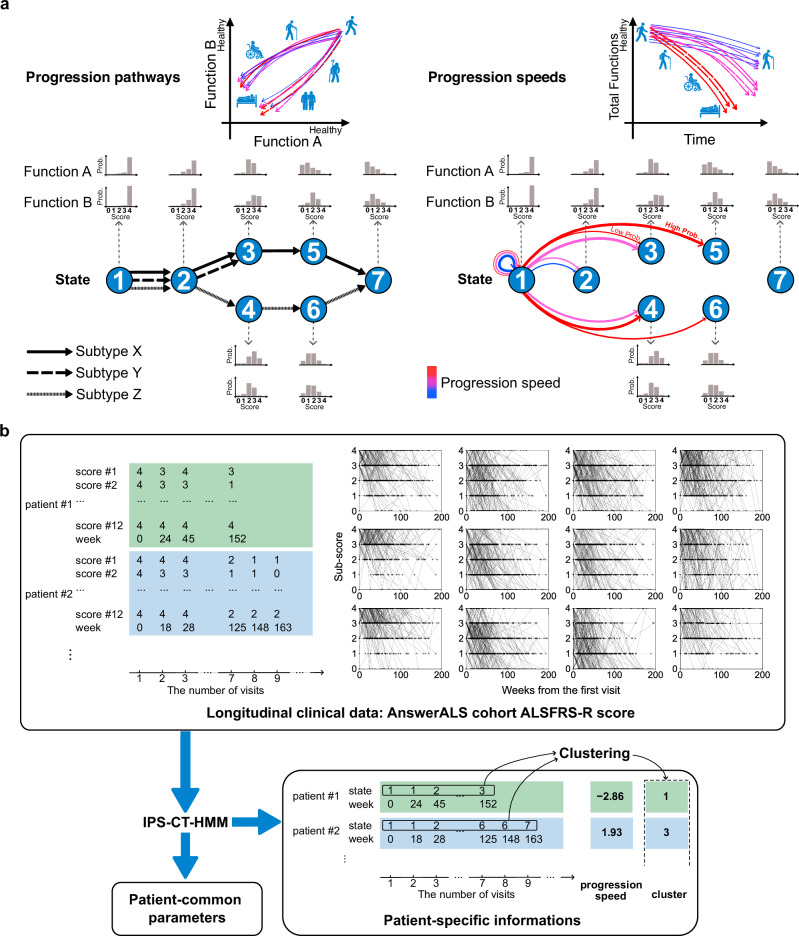
The concept of the present study. **a** The concept of the present study and the proposed model. The progression of chronic diseases, including neurodegenerative diseases, varies greatly among individuals. The heterogeneity of disease progression includes not only heterogeneity in the progression pathway, i.e., which functions deteriorate preferentially, but also heterogeneity in the speed of progression. The proposed individual-progression-speed continuous-time hidden Markov model (IPS-CT-HMM) can deal both heterogeneities. **b** Schematic diagram of the analysis flow with disease-progression speed and pathway analysis based on hidden Markov model (DiSPAH). Clinical longitudinal data, irregularly collected at the different number of time points for each patient, is input into IPS-CT-HMM for learning. The ALS Functional Rating Scale–Revised (ALSFRS-R) is a clinical functional score for ALS consisting of 12 questions scored from 0 to 4 points, divided into four domains. Each column represents questions from a different domain. IPS-CT-HMM estimates patient-common parameters defining potential disease progression states, along with patient-specific trajectories of disease progression states and disease progression speeds. By clustering the estimated trajectories, patient clusters (subtypes) with similar trajectories are obtained.

To tackle this challenge, it is critical to decompose the heterogeneity of disease progression into two orthogonal dimensions (Fig. 1a). The first is the progression pathway, which describes which functions deteriorate first and in what order. The second is the progression speed, which quantifies how quickly a patient moves through the stages of disease. These two axes of heterogeneity may arise from different biological mechanisms, but most previous studies have not clearly distinguished them. A framework that can disentangle these aspects is essential for capturing the full complexity of disease dynamics at the individual level.

Disease course is often conceptualized as transitions among discrete stages to support clinical interpretability, as in continuous-time multi-state survival models for irregular follow-up^17,18^, with latent-state extensions to accommodate noisy clinical observations^19–22^. In parallel, subject-specific time reparameterization approaches can orthogonalize an individual’s pace from the underlying trajectory^23^, but they have rarely been integrated with latent, discrete-state disease progression models.

ALS exemplifies a disease in which heterogeneity in both progression pathway and speed significantly contributes to clinical variability^11,24^. It is a neurodegenerative disorder in which patients gradually lose control of voluntary muscles due to the degeneration of motor neurons in the central nervous system^25^. While progression is typically rapid with an average life expectancy of approximately 3 years after diagnosis^24^, some patients survive for more than 10 years^26,27^. Long-term survival is more frequently observed in younger-onset and limb-onset patients^26,28^. ALS also shows phenotypic heterogeneity, including variation in symptom onset site and the pattern of functional decline^29–31^. Currently, no cure exists, and only a few drugs are approved that slightly slow disease progression. This wide variation complicates prognosis and hinders therapeutic development. A framework that separately models progression pathway and speed is thus essential for advancing ALS research and personalized care.

In this study, we developed a machine learning framework called DiSPAH (Disease-progression Speed and Pathway Analysis based on a Hidden Markov model) to jointly estimate progression pathway and progression speed at the individual level. DiSPAH employs a continuous-time hidden Markov model (CT-HMM)^19–22,32–34^, in which clinical features—observed at irregular hospital visits—are probabilistically generated from latent disease states. Transitions between these latent states capture the diversity of progression pathways. In this work, we focus on ALS Functional Rating Scale–Revised (ALSFRS-R) as a widely used longitudinal clinical measure in ALS and conceptually treat it as a downstream and noisy readout of an underlying, unobserved disease burden. We hypothesize that patients share a common set of latent disease states reflecting this burden. We adopt discrete latent states as a pragmatic and clinically interpretable choice in the absence of an established dynamical model of a continuous latent disease burden. To model individual differences in progression speed, we introduce a patient-specific speed parameter that time-scales transition rates. DiSPAH operationalizes this concept by learning shared latent states while allowing both patient-specific progression speed (via an individual speed parameter) and patient-specific realized pathways (via probabilistic branching transitions). We applied DiSPAH to longitudinal data from ALS cohorts^35,36^ to estimate each patient’s latent disease trajectory and progression speed. We further showed that these individualized dynamics were associated with baseline clinical features and that both progression speed and pathways could be predicted from data available at the first clinical visit. DiSPAH thus provides a powerful framework for uncovering heterogeneous disease progression across individuals.

Clinically, ALS progression is often summarized using measures such as the ALSFRS-R linear slope or baseline prognostic scores (e.g., ENCALS), which provide useful but coarse summaries of overall decline or survival risk. In contrast, DiSPAH leverages irregularly sampled longitudinal data to infer both a patient’s progression pathway (the domain-specific pattern of functional change) and progression speed (how rapidly latent disease states are traversed), thereby providing complementary information beyond a single slope or risk score. This separation may enable more interpretable stratification and improved forecasting of functional deterioration, supporting patient counseling and clinical trial design.

## Results

### A framework to analyze heterogeneous disease progression

We have developed a machine learning framework for analyzing longitudinal clinical data from patients with diverse disease progression pathways and speeds, which we call DiSPAH (Disease-Progression Speed and Pathway Analysis based on a Hidden Markov model). DiSPAH is based on HMM to model how patients’ symptoms evolve over time, as recorded in longitudinal clinical data. Observed symptoms (e.g., clinical scores) are assumed to be probabilistically generated from underlying unobservable, latent disease states. These disease states are discrete and evolve over time through probabilistic transitions. To handle irregular timing of hospital visits, DiSPAH adopts a continuous-time HMM (CT-HMM), which describes the transition probability depending on time interval between observations. To reflect individual differences in disease progression speed, DiSPAH introduces a patient-specific speed parameter, which proportionally scales transition probability for each patient. This means that patients with larger progression speed parameters have a higher probability of transitioning to a worse state than those with smaller parameters (Fig. 1a). We refer to this extended CT-HMM as the individual-progression-speed CT-HMM (IPS-CT-HMM).

By learning the time series data of the clinical scores at various time points from a population of patients with ALS, DiSPAH enables estimation of (i) a definition of the latent disease states that are represented by probabilistic distribution of clinical observations, (ii) individualized progression trajectories through the latent disease states, and (iii) progression speeds for each patient. Furthermore, clustering the inferred trajectories allows the identification of patient subgroups with similar sequences of the latent disease states (i.e., disease progression pathways) (Fig. 1b). Although the transition structure is shared, it is probabilistic and can branch, so different participants may realize different latent-state sequences. These pathway differences cannot be captured by the initial state and progression speed alone; therefore, we cluster estimated state trajectories to quantify pathway heterogeneity beyond baseline and speed. Detailed explanations of the proposed framework and the estimation algorithm are described in Methods.

### Application to longitudinal data from patients with ALS

**Fig. 2:**
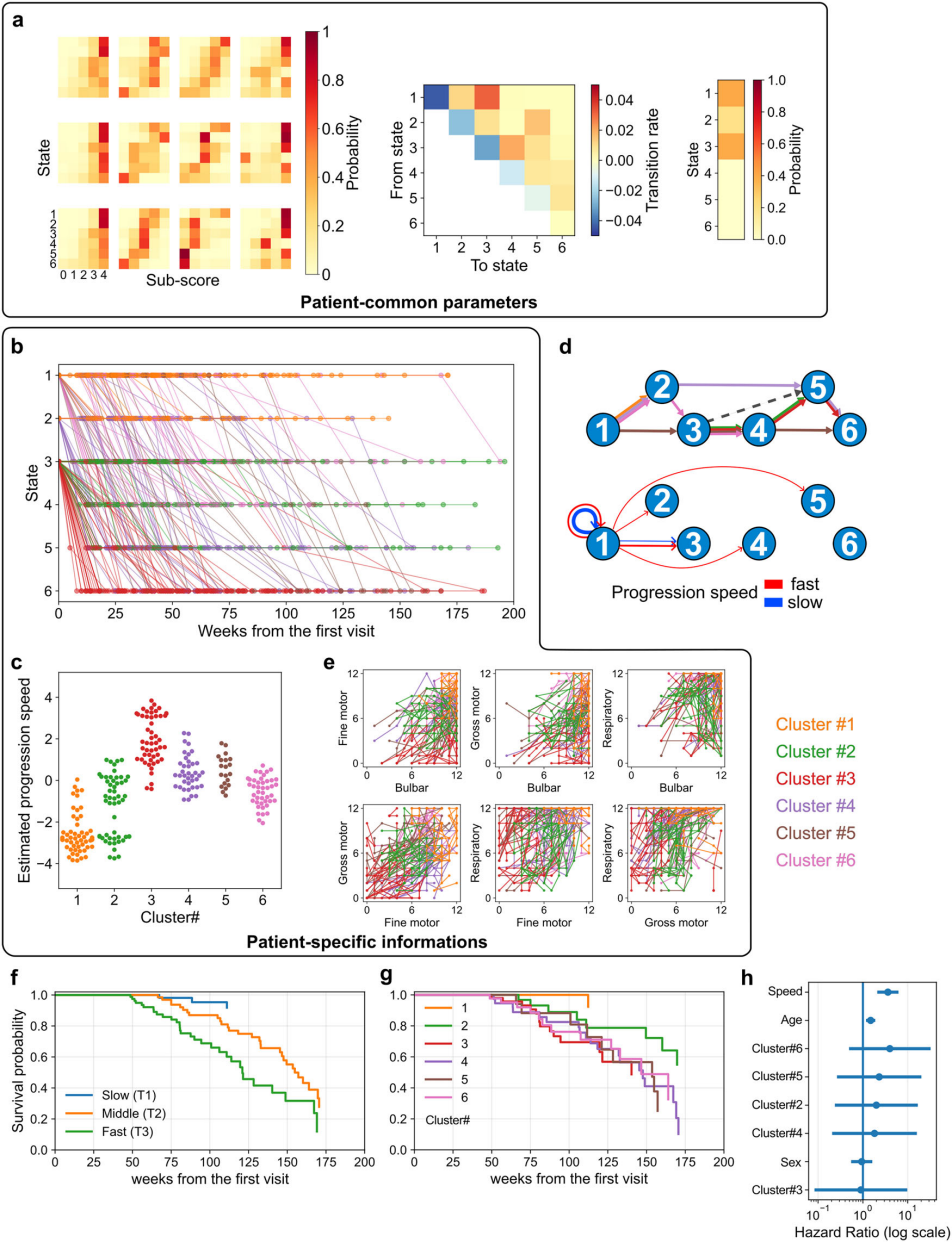
DiSPAH elucidated the heterogeneous disease progression in AnswerALS cohort. **a** Patients-common parameters estimated from the ALSFRS-R longitudinal data of AnswerALS cohorts. Emission probability matrices (left: the probability of observing a given score at each disease progression state), transition rate matrix (center: the proportion of transitions from one state to another), and initial state probabilities (right: the probability of being in each disease progression state at the start) were estimated. The emission probability matrices include 12 matrices for each question. Each column represents questions from a different domain. In the transition rate matrix, lower-triangular entries corresponding to disallowed backward transitions are masked to avoid visual confusion with terminal-state transitions. **b** The estimated patient-specific trajectories of disease progression states. **c** The estimated patient-specific disease progression speeds. **d** The schematic diagram of the estimated disease progression pathway (upper) and speed (bottom). In the pathway diagram, trajectories were drawn based on representative staying states within each cluster. A dashed line indicates a state transition that present in the transition rate matrix but is not included in the representative state transitions. In the speed diagram, “fast” represents the probability of state transition over 5 weeks with $v=1.5$, while “slow” represents that with $v=-0.5$. Transitions with a probability of 0.05 or higher are indicated by arrows. **e** Patient-specific trajectories in the domain total scores of AnswerALS cohort patients. In **b**–**e**, each color represents a patient cluster obtained by clustering the estimated progression trajectories. **f** Kaplan–Meier curves for overall survival stratified by estimated progression speed tertiles (Slow, Middle, Fast). Time is measured in weeks from the first ALSFRS-R assessment. **g** Kaplan–Meier curves for overall survival stratified by estimated progression clusters. **h** Multivariable Cox proportional-hazards model for overall survival including standardized progression speed, clusters and baseline clinical covariates (sex and age at symptom onset). Points denote hazard ratios (HR) and horizontal bars indicate 95% confidence intervals; the vertical line indicates HR = 1. Cluster #1 was used as the reference category.

We applied the developed DiSPAH to the longitudinal ALSFRS-R scores from AnswerALS cohort^35^. The ALSFRS-R is a widely used measure of functional status in patients with ALS^37^. It comprises 12 items spanning four functional domains—bulbar (questions 1–3), fine motor (questions 4–6), gross motor (questions 7–9), and respiratory (questions 10–12)—with each question scored from 0 (severe impairment) to 4 (normal function), yielding a total score range of 0–48 (higher scores indicate better function). We also report domain subtotals (0–12 per domain) to summarize longitudinal functional changes and to visualize which functional domains tend to decline preferentially in patients. For this analysis, we included only patients with at least four visits to the hospital. Given the well-documented heterogeneity in progression pathways and speeds by site of disease onset^25–28^, we restricted our analysis to patients presenting with limb-onset ALS. This subgroup comprised the largest proportion of cases in the cohorts, enabling more reliable estimation of model parameters. A total of 264 patients met these criteria and are included in the analysis (Supplementary Table 1). The EM algorithm was used to learn the ALSFRS-R time-series data (Supplementary Fig. 1), estimating the following three model parameters common to all patients: the emission probability matrix, which describes the probability of observing a certain score on a given question in each latent state (Fig. 2a, left), the transition rate matrix, which indicates transition probability from one latent state to another (Fig. 2a, center), and the initial state probability, which is the probability of being in a certain latent state at the start of tracking (Fig. 2a, right). The emission probability matrix shown in the left panel of Fig. 2a clarifies how different score values are distributed within each latent state. Specifically, for each latent state and each ALSFRS-R item, the model defines a categorical probability distribution over the possible observed scores (0–4). Thus, a latent state does not correspond to a single fixed observed score; rather, multiple score values can be observed within the same state, with their relative probabilities given by the learned emission probabilities. The estimated probabilities reflect the relative frequency distribution of score values within a state, although they are estimated from posterior-weighted observation counts across all observation time points rather than from hard state assignments. Throughout all analyses, we used *K* = 6 latent disease states, selected by patient-level cross-validation of likelihood (Methods). Since the patient’s disease condition is expected to worsen monotonically over time in ALS or other neurodegenerative diseases, we assumed that transitions of disease state occur in a one-way fashion. The learned latent states formed clinically interpretable stages with distinct expected ALSFRS-R domain profiles (Supplementary Table 2). From the ALSFRS-R time-series data, we estimated not only patient-common parameters, but also patient-specific disease progression speeds. The estimated progression speeds appeared correlated with the slope of the regression line to the total score of ALSFRS-R^35^, but a distinct difference between them was observed in a certain number of patients (Supplementary Figs. 2, 3). We additionally evaluated goodness-of-fit using reconstruction-based diagnostics. Using posterior state probabilities computed during model fitting and the learned emission probabilities, the model demonstrated high agreement between observed and reconstructed total ALSFRS-R scores (R² = 0.801; Supplementary Table 3). Because the patient-specific progression-speed parameter is multiplicatively coupled with the transition rate matrix, the absolute scale of progression speed is not uniquely identifiable; importantly, the clinically relevant quantity is the relative progression speed across patients. To assess robustness of these relative estimates, we conducted two sensitivity analyses. First, we compared patient-specific progression speeds obtained under joint estimation of the transition rate matrix and speeds with patient-specific progression speeds obtained under re-estimation of speeds while holding a pre-estimated transition rate matrix fixed (Supplementary Fig. 4). As a result, relative speed estimates were highly consistent (Pearson’s correlation $r=0.986,{p} < 0.0001$). Second, we evaluated sensitivity to the scale of the transition rate matrix by perturbing off-diagonal transition rates by $\pm 20 \% \,$(recomputing diagonal entries to preserve row-sum constraints) and re-estimating patient-specific progression speeds with the perturbed $Q$ fixed (Supplementary Fig. 4). Relative speed estimates remained highly stable (Pearson’s correlation $r=0.962,{p} < 0.0001$ for + 20%, and $r=0.999,{p} < 0.0001$ for −20%).

Using the estimated parameters, we further inferred patient-specific trajectories of latent states. By clustering these trajectories, we obtained six clusters of patients who showed similar disease progression pathways (Fig. 2b). While progression speeds tended to be localized within clusters, there was notable overlap in the distributions across clusters, suggesting that even patients with similar progression speeds may follow different latent-state progression pathways (Fig. 2c). This indicates that the proposed method can separately analyze the diversity of disease progression pathways and the diversity of progression speeds (Fig. 2d). To aid clinical interpretation, we provide illustrative patient pairs with similar baseline total ALSFRS-R and the same trajectory cluster but markedly different inferred progression speeds, showing divergent subsequent decline in total ALSFRS-R (Supplementary Fig. 5). We additionally illustrate an example in which two patients have similar total-score slopes yet substantially different estimated speeds (Patient D and F, Supplementary Fig. 5b, c).

Each cluster exhibited characteristic disease progression pathways. Cluster #1 included patients with slow disease progression speed, remaining in state 1 and 2. These patients showed gradual deterioration in motor function, but little decline in bulbar or respiratory function. Cluster #2 patients had motor function decline from the start of follow-up, but the progression speed was similar to that of Cluster #1, and they did not reach a state of complete functional deterioration. In contrast, Cluster #3 patients began at a state similar to those in Cluster #2 but exhibited rapid progression, with deterioration predominantly in motor function, eventually reaching latent state 6 (i.e., final ALS state). Cluster #4 was characterized by an unusual pattern in which gross motor function deteriorated earlier than fine motor function. Cluster #5 showed a progression speed comparable to Cluster #4 but with the opposite pattern: fine motor function declined earlier than gross motor function. Cluster #6 began with functional levels similar to Cluster #1 but experienced a slightly faster and more generalized functional decline overall (Fig. 2e, Supplementary Fig. 6).

### Simulation-based validation of patient-specific information estimation

To assess how well the proposed IPS-CT-HMM can recover progression speed and latent pathways under realistic irregular sampling, we conducted simulation-based validation. We generated synthetic longitudinal ALSFRS-R score trajectories from the IPS-CT-HMM generative process, using the patient-common parameters estimated from the AnswerALS cohort (i.e., the emission probability matrix, the transition rate matrix and the initial state probability), while randomly sampling patient-specific progression speeds for each simulated patient. We varied the mean visit interval while holding the total follow-up duration fixed. Specifically, we considered three conditions (mean interval = 10, 20, 40; total follow-up = 200 weeks), where the number of observations per patient was automatically adjusted to match the fixed follow-up window and per-patient visit schedules were randomly jittered while preserving the exact end time. For each condition, we generated multiple replicate datasets and re-fit the IPS-CT-HMM using the same estimation procedure as in the analyses of real-world data and quantified recovery of (i) progression speed (correlation between true and estimated speeds) and (ii) sequence of latent states (observation-point-wise agreement of true and estimated state sequences). We found that speed estimation remained stable (Pearson’s correlation *r* = 0.81–0.83), and pathway recovery was high (accuracy was 0.84 at mean interval 10 and 0.97–0.98 at mean intervals 20–40) across all conditions, supporting that the model can reliably infer its key latent quantities under irregularly sampled longitudinal data (Supplementary Fig. 7).

### Survival and functional milestone endpoints analysis using speed and pathways

To place DiSPAH-derived latent dynamics in a clinically interpretable framework, we evaluated time-to-event outcomes using weeks from the first ALSFRS-R assessment as the time origin. We examined overall survival and domain-specific functional deterioration milestones derived from ALSFRS-R.

Kaplan–Meier analyses stratified by progression speed (tertiles) showed clear separation in survival probability (Fig. 2f). Pathways also stratified survival, with Cluster #1 and Cluster #2 exhibiting survival patterns distinct from the remaining clusters (Fig. 2g). In Cox multivariable proportional-hazards models including standardized speed, cluster indicators (dummy variables), and baseline clinical covariates (sex and age at symptom onset), progression speed was associated with increased hazard (Fig. 2h, HR = 3.61, 95% CI = 2.09–6.23, $p < 0.0001$), providing a clinically interpretable mapping of the estimated speed parameter to global disease aggressiveness.

We next evaluated the relationship of speeds and pathways with domain-specific functional deterioration milestones. We computed ALSFRS-R domain subtotals—Q1–Q3, Q4–Q6, Q7–Q9, and Q10–Q12—and analyzed time to reach severe impairment (domain subtotal ≤6) for each domain. Pathways stratified the timing of these milestone events (Supplementary Fig. 8), indicating that pathways capture heterogeneity in the domain-specific pattern and timing of severe functional deterioration beyond overall progression speed. Consistently, in Cox multivariable model analysis, standardized speed remained associated with risk, while pathways provided complementary prognostic stratification for clinically interpretable endpoints (Supplementary Fig. 8), supporting the added clinical value of the pathway classification beyond speed alone.

### Verification of the estimated disease states and clusters using a larger cohort

**Fig. 3:**
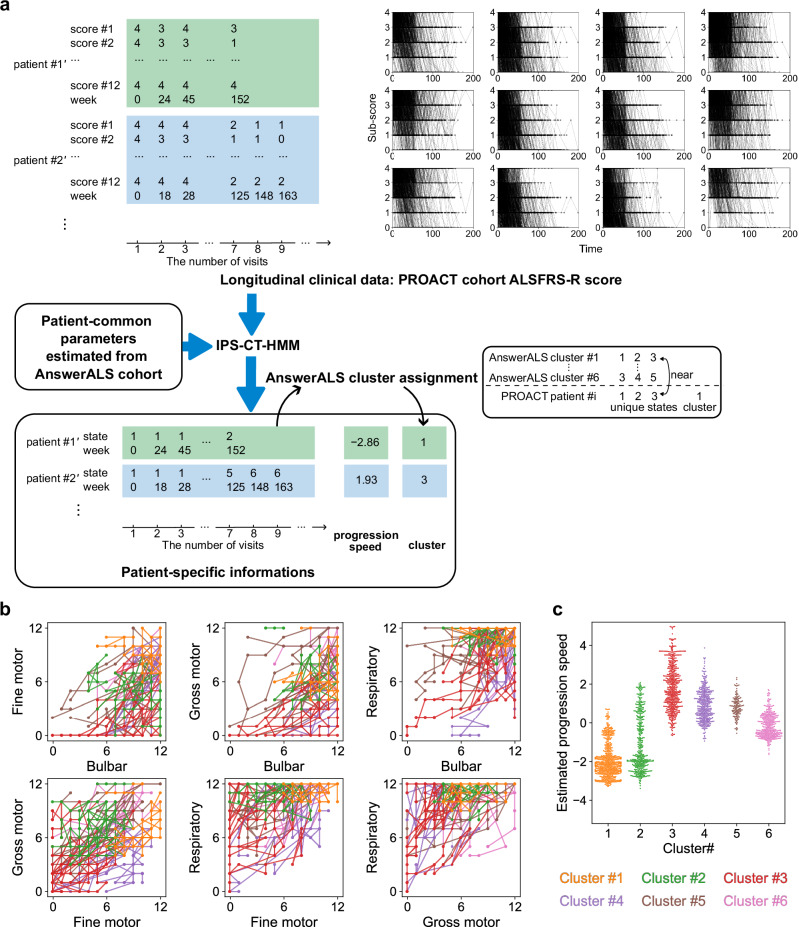
Verification of the elucidated heterogeneous disease progression in a larger cohort. **a** Schematic diagram of the analysis flow of PRO-ACT cohort data using disease progression states estimated from AnswerALS cohort data. Longitudinal ALSFRS-R data is learned using IPS-CT-HMM. At this stage, patient-common parameters obtained from the AnswerALS data are provided to the model as prior information, and patient-specific progression trajectories and progression speeds are estimated. PRO-ACT cohort patients are classified into respective clusters by referencing the representative disease progression states of each cluster obtained from AnswerALS data. **b** Patient-specific trajectories in the domain total scores of 20 PRO-ACT cohort patients randomly selected from each cluster. **c** Distribution of the estimated patient-specific progression speeds for each cluster.

To assess the universality of the probabilistic definition of ALS progression states and progression pathway clusters obtained from the AnswerALS cohort, we applied DiSPAH to the larger PRO-ACT cohort data (Fig. 3a). Following the same criteria used in AnswerALS to select patients, 2,565 individuals were included in the analysis (Supplementary Table 1). When training the IPS-CT-HMM model in the PRO-ACT cohort data, we incorporated patient-common parameters, i.e., the emission probability matrix, the transition rate matrix and the initial state probability, previously estimated from the AnswerALS cohort as prior information and used them without updating, allowing the model to infer only patient-specific information for the PRO-ACT data. We observed similarly high agreement between observed and reconstructed total ALSFRS-R scores in PRO-ACT as in AnswerALS (R² = 0.800; Supplementary Table 3). Furthermore, for each patient in the PRO-ACT cohort, we compared the estimated state transitions with those of AnswerALS cohort patients and classified them into the AnswerALS cohort patient cluster with the closest sequence of staying states. As a result, we obtained that the distribution of functional decline pathways (Fig. 3b) and disease progression speeds (Fig. 3c) among patients within each cluster in the PRO-ACT cohort closely resembled those in the AnswerALS cohort. This consistency suggests that our proposed DiSPAH framework captures cohort-invariant features of ALS progression, rather than cohort-specific ones. However, one notable exception was observed: patients in Cluster #2 of the AnswerALS cohort showed a decline in respiratory function, whereas such a pattern was not observed in the corresponding cluster of PRO-ACT cohort (Fig. 3b, Supplementary Fig. 9).

Following the validation of disease states and clusters in PRO-ACT, we next asked whether patient-specific progression speed provides additional benefit for forecasting ALSFRS-R trajectories beyond a non-personalized CT-HMM. We therefore compared IPS-CT-HMM with a uniform-speed CT-HMM in a prediction analysis on PRO-ACT. We trained the two models separately on AnswerALS and carried forward the corresponding patient-common parameters for each model to PRO-ACT. For each PRO-ACT subject, we conditioned on the first three ALSFRS-R observations and evaluated prediction of ALSFRS-R scores occurring within one year after the third observation. Predictions were computed using soft state posteriors at the third observation and continuous-time propagation of the latent-state distribution. In the IPS-CT-HMM, a patient-specific progression speed was estimated from the first three observations and used to scale the continuous-time transition rates during forecasting, whereas the uniform-speed CT-HMM fixed the speed parameter across subjects. Performance was assessed using point-prediction error for total ALSFRS-R (MAE/RMSE/R²) and predictive log-likelihood. In PRO-ACT patients with baseline total ALSFRS-R ≥ 36 (1798 patients), the IPS-CT-HMM outperformed the uniform-speed CT-HMM in one-year-ahead trajectory prediction from the first three observations. Total-score prediction errors were lower under IPS-CT-HMM (MAE 4.04 vs 4.17; RMSE 5.43 vs 5.56), with higher variance explained (R² 0.398 vs 0.368) (Supplementary Fig. 10). Probabilistic forecasting performance was also substantially improved, as reflected by higher predictive log-likelihood. Together, these results indicate that incorporating patient-specific progression speed yields measurable gains over a standard non-personalized CT-HMM for predicting ALSFRS-R trajectories in PRO-ACT.

### Association between estimated progression speed and ALS-related genetic information

**Fig. 4:**
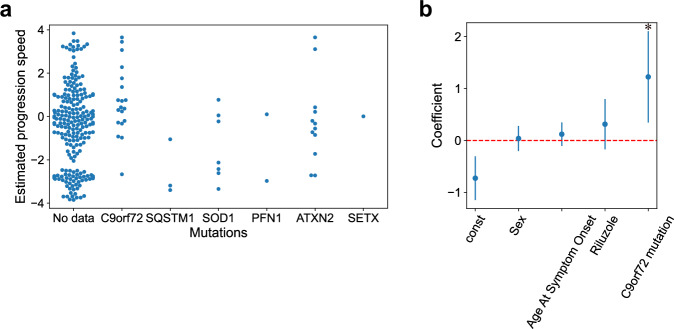
Association between estimated disease progression speed and ALS-related mutations. **a** Relationship between ALS-related gene mutations and estimated progression speeds. **b** Coefficients estimated by multivariable regression with the presence or absence of C9orf72 mutation as the main explanatory variable and the estimated progression speeds as the target variable, adjusted for sex, age at symptom onset, and riluzole use. The error bars indicate 95% confidence intervals.

We next investigated the association between the estimated disease progression speed and known ALS-related genetic mutations. The AnswerALS cohort data contains information on these mutations identified in patients (Fig. 4a). Among these, C9orf72, ATXN2, and SOD1 mutations were identified in a sufficient number of patients and were therefore selected for further analysis. We performed a multivariable regression analysis to predict the estimated progression speed by the presence and absence of each gene mutation as explanatory variables, and also covariates of gender, age at onset, and prior exposure to riluzole^28,38^ (Supplementary Fig. 11a–c). The analysis revealed that only the C9orf72 mutation had a statistically significant positive regression coefficient (Fig. 4b, $p < \,0.01$), meaning that patients with the C9orf72 mutation exhibit faster disease progression compared to those without it. The finding is consistent with the previous report linking C9orf72 mutation to shorter survival duration in ALS^39,40^. The patients who have SOD1 mutation appeared to have relatively slow progression, but no statistically significant differences were observed (*p* = 0.197). No significant associations were also found in the patients with ATXN2 mutations (Supplementary Fig. 11d, e, ${p}=\,0.800$). We further assessed the continuous association between C9orf72 repeat expansion length (log10-transformed) and the estimated progression speed. No clear linear relationship was observed (Pearson’s correlation ${r}=\,0.136,{p}=\,0.0982$) and no significant association was observed in the regression analysis (Supplementary Fig. 11f, g, ${p}=\,0.110$).

### Discovering biological associations of disease progression speed using patient iPSC-derived motor neurons

**Fig. 5:**
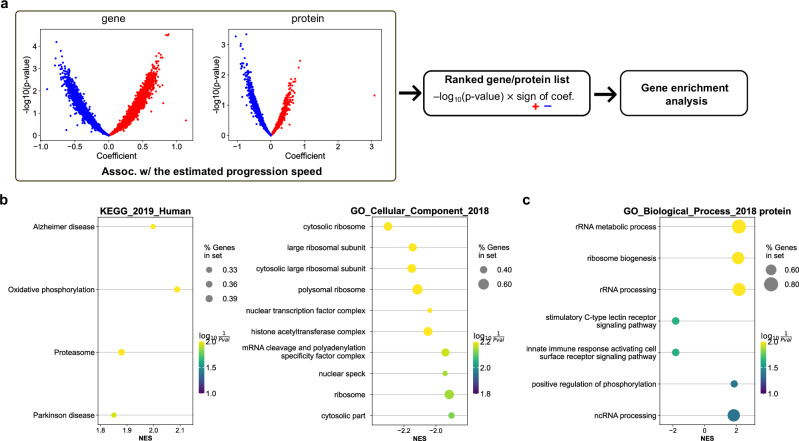
Analysis of the association between gene/protein expression in patient-derived iPSC-MNs and estimated disease progression speed. **a** The workflow in Gene enrichment analysis. First, perform multivariable regression with specific gene/protein expression levels as the primary explanatory variables and estimated progression speeds as the target variables, then rank the genes/proteins based on the *p*-values and the estimated coefficient signs. Use this ranking to perform GSEA analysis. **b** Examples of gene groups exhibiting significant fluctuations in relation to estimated progression speed, obtained from GSEA analysis in the transcriptome. **c** Those obtained from the proteome.

To explore biological factors associated with the progression speed of ALS, we evaluated the relationship between genome-wide gene expression in motor neurons derived from patients. In the Answer ALS dataset, induced pluripotent stem cells (iPSCs) were established from patients, differentiated into motor neurons (iPSC-MNs), and subjected to transcriptome and proteome data^35^ (Fig. 5a).

To examine the association between the DiSPAH-estimated disease progression speed and individual gene expression in the motor neurons, we performed multivariable regression analysis for each gene, in which disease progression speed was set as the target dependent variable, and expression level of each gene as explanatory variable (*n* = 84). Sex, age at onset, riluzole exposure status and C9orf72 mutation status were included as covariates in all models. No single gene was identified as significantly influencing disease progression speed (Fig. 5a). To explore broader functional trends, we ranked genes based on *p*-values and regression positive or negative regression coefficients and conducted gene enrichment analysis using GSEA^41,42^. In GSEA, the analysis targeted KEGG pathways and Gene Ontology (GO) categories: Biological Process (BP), Cellular Component (CC), and Molecular Function (MF). In the KEGG pathway analysis, significant upregulation was observed in pathways related to neurodegenerative diseases such as Alzheimer’s disease and Parkinson’s disease, as well as in pathways related to oxidative phosphorylation and proteasome function. In GO CC analysis, we observed downregulation of gene clusters associated with mature ribosomes, such as polysomes (Fig. 5b, Supplementary Fig. 12a, b).

We also applied a similar multivariable regression analysis to the proteomic data (*n* = 59), evaluating the association between protein expression and the DiSPAH-estimated disease progression speed. As with the transcriptomic data, no significant association with a single protein was observed (Fig. 5a). However, GSEA analysis revealed upregulation of protein groups related to ribosome biogenesis in GO BP category (Fig. 5c, Supplementary Fig. 12c).

The results suggest the following pathophysiological cascade: abnormal protein aggregation leads to the suppression of global translation, triggering a compensatory up-regulation of ribosome biogenesis, resulting in the accumulation of immature ribosomal precursors. In parallel, cells boost oxidative-phosphorylation pathways to secure the ATP required for proteasome-mediated clearance of mis-folded or surplus proteins. Mitochondria stressed under this high-flux state leak electrons, raising reactive-oxygen-species (ROS) levels. The combined burden of translation arrest, proteostasis overload, and oxidative stress is hypothesized to accelerate motor-neuron damage, thereby amplifying disease progression through a vicious cycle of cellular dysfunction.

### Prediction of disease progression speed and pathway based on data available at the initial time point

**Fig. 6:**
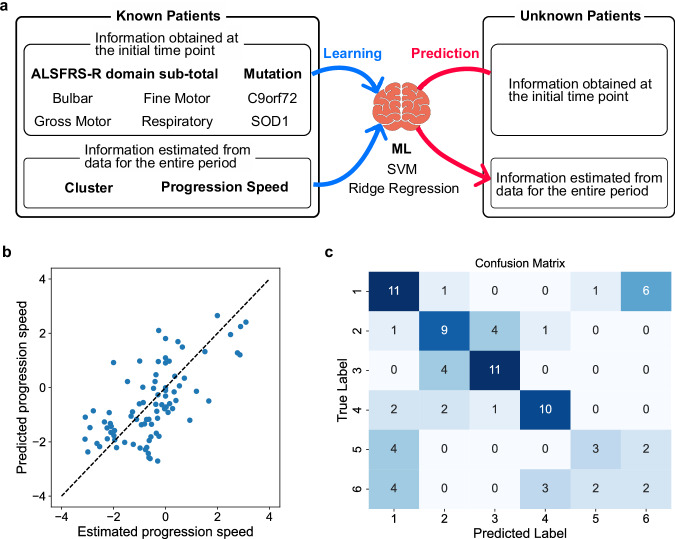
Prediction of disease progression speed and clusters based on information available at the start of tracking. **a** Analysis flow for predicting progression clusters and progression speeds based on information available at the initial time point for the follow-up. **b** The result for progression speed predictions. The progression speeds predicted from initial information and the progression speeds estimated from the whole period of data match on the dotted line. **c** The result for progression cluster predictions.

Finally, we evaluated the feasibility of predicting patient-specific progression speed and progression-trajectory cluster, which were estimated from longitudinal data by DiSPAH, based on information available at the start of follow-up. Specifically, we used the following features as input variables for prediction: (i) the subtotal score of ALSFRS-R for each functional domain (0–12 for Bulbar, Fine motor, Gross motor, and Respiratory functions), and (ii) the presence or absence of C9orf72 and SOD1 mutations, both of which were selected based on our multivariable genetic analyses and the GSEA results obtained from iPSC-MN omics data. These features were used as input variables, and the previously estimated progression speed or cluster labels were used as output variables. The relationships between inputs and outputs were learned by ridge regression (for progression speed regression) and support vector machine (SVM) with linear kernel (for cluster classification). To evaluate predictive performance, we applied leave-one-out patient-level cross-validation. In each iteration, the model was trained on a dataset excluding one patient, and the trained model was used to predict the progression speed or cluster label of the excluded patient. This process was repeated for all patients, and the overall prediction accuracy was evaluated (Fig. 6a).

The trained regression model showed reasonable performance in predicting progression speed, particularly for patients showing very slow and very fast estimated progression speeds. The mean squared error was 1.28, and the coefficient of determination was 0.34 (Fig. 6b). For progression cluster prediction, we also used the predicted progression speed as input for SVM in addition to the information available at the start of follow-up. As a result, we were able to correctly predict disease progression clusters with approximately 54.8% accuracy (Fig. 6c). Among the clusters, clusters #2 and #3, as well as clusters #5 and #6, showed relatively similar progression patterns. When these pairs of clusters were combined as broader clusters, the prediction accuracy improved to approximately 69.0%, indicating that the proposed method can effectively distinguish major progression trends (Fig. 6c). Gene expression levels in patient iPSC-derived motor neurons did not contribute to predicting progression speeds or progression pathway clusters (Supplementary Fig. 13). The results suggest that the proposed method may provide useful prognostic information at an early stage after onset.

To clarify the practical utility of DiSPAH relative to established ALS prognostic tools, we performed a benchmarking analysis against an ENCALS-like^43^ Cox proportional hazards model and evaluated the additional contribution of the predicted DiSPAH-derived features. We compared the outcome predictive performance of

- Model A (ENCALS-like): baseline predictors including pre-baseline ALSFRS-R slope proxy (ΔFRS)
- Model B1: Model A + predicted speed
- Model B2: Model A + predicted cluster (pathway)
- Model B3: Model A + predicted speed + predicted cluster
- Replacement models (R1–R3): using predicted speed/cluster instead of ΔFRS

Because the present study focuses on limb-onset patients, site of onset was excluded from the ENCALS-like predictors^43^. In Answer ALS, clinical timing variables (symptom onset, diagnosis, and death) are recorded as relative days from the first ALSFRS-R observation (day0), and we therefore used day0 as the baseline time origin. We evaluated discrimination using patient-wise 5-fold cross-validated concordance index (C-index).

We first assessed time-to-death (censoring at the subject’s last ALSFRS-R observation) (Table 1). Model A achieved C-index of 0.768. Adding predicted speed (Model B1) reduced performance (C-index: 0.730), whereas adding predicted pathway cluster (Model B2) yielded a small but consistent improvement (C-index: 0.773). Adding both predicted speed and cluster (Model B3) did not improve performance (C-index: 0.718). Replacement models using predicted speed and/or cluster in place of ΔFRS degraded discrimination. These results indicate that for survival prediction, ENCALS-like predictors capture most of the prognostic signal in this setting, with DiSPAH pathway information providing a modest additional benefit, while predicted speed is not a substitute for ΔFRS.

**Table 1:** Benchmark comparison of ENCALS-like Cox models and DiSPAH-derived features

Model	Mortality	Bulbar ≤ 6 (Q1-3)	Fine motor ≤ 6 (Q4-6)	Gross motor ≤ 6 (Q7-9)	Respiratory ≤ 6 (Q10-12)
A0	0.725 ± 0.043 (*n* = 5)	0.709 ± 0.031 (*n* = 5)	0.666 ± 0.035 (*n* = 5)	0.600 ± 0.044 (*n* = 5)	0.752 ± 0.028 (*n* = 5)
A	0.768 ± 0.037 (*n* = 5)	0.719 ± 0.057 (*n* = 5)	0.698 ± 0.055 (*n* = 5)	0.629 ± 0.024 (*n* = 5)	**0.763** ± **0.048** (*n* = 5)
B1	0.730 ± 0.245 (*n* = 5)	0.672 ± 0.061 (*n* = 3)	0.834 ± 0.037 (*n* = 5)	0.635 ± 0.107 (*n* = 5)	0.717 ± 0.095 (*n* = 5)
B2	**0.773** ± **0.046** (*n* = 5)	**0.739** ± **0.053** (*n* = 5)	0.711 ± 0.059 (*n* = 5)	0.646 ± 0.030 (*n* = 5)	0.762 ± 0.052 (*n* = 5)
B3	0.718 ± 0.196 (*n* = 5)	0.506 ± 0.075 (*n* = 3)	0.854 ± 0.034 (*n* = 5)	0.680 ± 0.120 (*n* = 5)	0.586 ± 0.152 (*n* = 5)
R1	0.672 ± 0.161 (*n* = 5)	0.389 ± 0.104 (*n* = 3)	0.824 ± 0.068 (*n* = 5)	0.681 ± 0.105 (*n* = 5)	0.749 ± 0.103 (*n* = 5)
R2	0.742 ± 0.046 (*n* = 5)	0.732 ± 0.038 (*n* = 5)	0.692 ± 0.033 (*n* = 5)	0.614 ± 0.057 (*n* = 5)	0.748 ± 0.026 (*n* = 5)
R3	0.709 ± 0.135 (*n* = 5)	0.256 ± 0.129 (*n* = 3)	**0.860** ± **0.054** (*n* = 5)	**0.739** ± **0.091** (*n* = 5)	0.669 ± 0.091 (*n* = 5)

Cross-validated C-index values (mean ± SD) are shown for mortality prediction and domain-specific ALSFRS-R milestone endpoints (first time domain subtotal ≤6; censoring at last observation). Analyses were restricted to limb-onset subjects; bulbar onset was therefore excluded from the ENCALS-like predictors.

Model definitions:

A0: baseline covariates without ΔFRS

A: ENCALS-like baseline including ΔFRS

B1: A + predicted speed

B2: A + predicted cluster (pathway)

B3: A + predicted speed + predicted cluster

R1: A0 + predicted speed (ΔFRS replaced by predicted speed)

R2: A0 + predicted cluster

R3: A0 + predicted speed + predicted cluster

For some bulbar milestone models, fewer than 5 folds were valid due to insufficient admissible pairs in the test split (n shown).

DiSPAH is designed to characterize longitudinal functional decline and pathway-specific patterns, which are not directly summarized by a single survival risk score. We therefore extended the benchmarking analysis to clinically meaningful functional milestones defined as the first time an ALSFRS-R domain sub-score fell to ≤6: bulbar (Q1–3), fine motor (Q4–6), gross motor (Q7–9), and respiratory (Q10–12), with censoring at the last ALSFRS-R observation. In this analysis, DiSPAH-derived features demonstrated domain-dependent added value (Table 1). For fine motor milestones, incorporating predicted speed and predicted cluster in place of ΔFRS yielded substantial gains (Model A: 0.698 → R3: 0.860), and similar improvements were observed for gross motor milestones. Bulbar milestones improved primarily with predicted cluster (Model A: 0.719 → Model B2: 0.739), whereas respiratory milestones were already well predicted by the ENCALS-like baseline model (Model A: 0.763) with little additional benefit from predicted speed/cluster. Together, these results suggest that DiSPAH offers complementary clinical value by enabling prediction of functional deterioration, which may be potentially relevant for counseling, rehabilitation planning, and trial endpoint selection.

## Discussion

We proposed DiSPAH, a modeling framework combining IPS-CT-HMM and trajectory clustering to dissect heterogeneity in ALS progression. Applied to the AnswerALS cohort, DiSPAH estimated latent disease states, individual progression speeds, and stratified patients into six distinct trajectory clusters. These findings generalized to the PRO-ACT cohort. Clinically, both progression speed and pathways were associated with time-to-event outcomes, stratifying overall survival and the timing of severe domain-specific functional deterioration milestones defined based on ALSFRS-R. A C9orf72 mutation was linked to faster progression, and multi-omics analysis of iPSC-MNs revealed molecular signatures of disrupted proteostasis and oxidative stress in fast progressors. Moreover, progression speed and cluster identity could be partially predicted from initial clinical and genetic information, and the predicted DiSPAH-derived features were useful for prognosis of functional deterioration complement with ENCALS-like predictors, highlighting the potential of DiSPAH for early prognosis and personalized trial stratification in ALS.

Longitudinal tracking of clinical scores, biomarkers, and neuroimaging has enabled data-driven stratification of disease progression in ALS and other neurodegenerative disorders^8,44,45^. Recent machine learning-based approaches have identified subgroups of patients with distinct progression trajectories, shedding light on disease heterogeneity. In ALS, clustering of longitudinal ALSFRS-R data reveals heterogeneous progression subtypes^11^. Recent approaches applying Gaussian mixture processes^8^ or triclustering^11^ to longitudinal data from the ALSFRS-R revealed heterogeneous subtypes of progression. Similar machine learning approaches have been applied to other neurodegenerative diseases. In Alzheimer’s disease, subtypes have been identified using event-based models^4,46,47^ and Bayesian clustering on biomarker and brain imaging data^48^. In Parkinson’s disease, subtypes have been identified by latent time joint mixed-effects models and deep generative model-based clustering^49^ applied to clinical scores and biomarkers. Among these previous studies, subtypes exhibiting characteristic progression speeds have been reported^49^. Unlike these existing methods, the proposed method explicitly separates the estimation of progression speeds from the clustering of progression pathways, enabling the identification of factors influencing progression speeds. This offers a different perspective to previous methods, and may promote a more detailed understanding of disease mechanisms.

In this study, we defined disease progression speed as a parameter that modulates the probability of state transitions occurring over a given time interval in a continuous-time hidden Markov model (CT-HMM)^19,20^, and estimated it from clinical data with irregular observation intervals. A widely used method for assessing ALS progression is to just fit a straight line to the total ALSFRS-R score over time and use the slope as an index of disease speed^50,51^. This conventional linear-fit approach assumes that overall functional decline in ALS proceeds uniformly and linearly across all domains, which rarely reflect actual disease dynamics^8^. In practice, however, symptom often worsen sequentially in specific domains or involves multiple functional systems progressing at different times, resulting in non-linear decreases in the total score. Moreover, in actual data, clinical scores may occasionally improve, leading to incorrect estimates when using linear fitting. By contrast, our probabilistic model-based approach is better suited to capturing these real-world complexities.

When comparing the disease progression speeds estimated by DiSPAH with the ALSFRS-R progression slope recorded in the database, we observed a moderate correlation. However, a certain number of patients showed relative difference between the two measures (Supplementary Fig. 2). Discordant cases were enriched for patients with lower baseline total ALSFRS-R, and a sharp decline in a small number of scores between the first two observations often coincided with a high DiSPAH speed estimate. This helps explain why DiSPAH may classify such cases as fast progressors even when the linear slope is attenuated by plateaus. Although survival information was available for only a limited subset, age at death tended to be slightly lower in the discordant group (Supplementary Figs. 2, 3). ALSFRS-R slopes can vary substantially under irregular follow-up and may occasionally be positive, likely reflecting measurement variability and sampling effects rather than true disease reversal. Because our IPS-CT-HMM enforces monotonic latent progression, such fluctuations are treated as observation-level noise, and the estimated progression-speed parameter has the potential to serve as a complementary, model-based indicator of overall progression speed.

We identified six states and six clusters representing distinct disease progression pathways in the analysis of limb-type patients with ALS. These states and clusters captured key aspects of progression, including: (i) how rapidly bulbar and respiratory functions declined relative to motor deterioration, (ii) whether fine-motor or gross-motor abilities deteriorated first, and (iii) whether a given function was already impaired at baseline or became completely lost by the end of follow-up. Regarding the third point, if our modeling and clustering framework could incorporate information beyond the observation window, it may be possible to stratify patients more appropriately. Clinically, limb-onset ALS is often categorized into upper limb-predominant and lower limb-predominant types^10,52^. The order of fine and gross motor decline observed in our results may reflect these known clinical subtypes.

As a limitation of the proposed model, there is a complementary relationship between the speed parameter and the transition rate matrix. Hence, the absolute value of the estimated progression speed may depend on the overall scale of the transition rates. However, when applied to real-world clinical data, an absolute notion of progression speed does not exist and is meaningful only in relative terms. Accordingly, this study focused on estimating relative progression speed across patients. We estimated the progression speed using a prior Gaussian distribution with a mean of zero. The choice of the hyperparameters value requires further investigation, and alternative methods such as the method of Lagrange multiplier can be considered to regularize estimation. A detailed examination of the constraint setting method and calculation method is a future task. Also, the systematic differences in ALSFRS-R administration across sites/countries and between in-clinic versus remotely self-administered assessments are not explicitly modeled in the current framework. Extending the model in hierarchical manner for incorporating such site- and mode- specific effects may improve robustness and cross-cohort comparability.

We restricted model training to patients with limb-onset ALS, and thus the current estimates may not generalize to bulbar-onset or other phenotypes without modification. The site of onset is a major, well-established source of heterogeneity in ALS, and including markedly different onset phenotypes in a single finite-state model risks conflating known phenotype differences with the latent heterogeneity that DiSPAH aims to capture from longitudinal trajectories. Moreover, given the limited descriptive capacity of a finite-state progression model and finite sample sizes for stable parameter estimation, narrowing the scope to a more homogeneous subgroup improves reliability. As a future extension, a multi-dimensional (domain-specific) latent state and speed formulation would be a promising direction to better accommodate phenotype-specific and domain-asynchronous progression.

Related to patient selection, selection bias and attrition are important limitations of longitudinal cohorts and may influence the estimated results. Future work should evaluate robustness to such biases and pursue further validation in diverse cohorts. The functional milestone analyses also have a limitation: because the true threshold-crossing time is only known to occur between visits, these endpoints are interval-censored. Accordingly, treating the first observed threshold crossing as the event time may introduce bias, particularly under long interval or irregular follow-up. In addition, DiSPAH does not explicitly include death as a terminal state; therefore, potentially informative censoring—particularly in heterogeneous trial-derived datasets such as PRO-ACT, where follow-up may be truncated around death or tracheostomy/ventilation—could affect observed trajectories (including respiratory items) and partly contribute to between-cohort differences (e.g., observed for Cluster #2 in this study). Extending the model with an absorbing death state^3,18^ is an important direction for future work.

In the proposed method, clusters were identified by hierarchical clustering based on estimated patient-specific state trajectories. An alternative approach to stratifying disease progression in patients involves incorporating subtypes directly as hierarchical latent variables within the probabilistic model and estimating them jointly with other parameters^8^. While such methods are promising, as they allow for comprehensive model-based clustering, they rely on the assumption of the existence of discrete subtypes, which may obscure continuous spectrum-like heterogeneity. For this reason, we selected to use post-hoc clustering in this study. Although this post-hoc pipeline consists of multiple steps, each was chosen to support pathway-focused clustering under the practical constraints of ALSFRS-R longitudinal data rather than to introduce additional modeling assumptions. In particular, time-aligned trajectory distances (DTW) and distribution-aware comparisons (Wasserstein) enable similarity assessment that is less sensitive to unequal follow-up schedules and phase shifts in functional decline. Nonetheless, we acknowledge that post-hoc clustering involves design choices. An alternative approach involving the hierarchical assumption of continuous “disease progression pathway” variables rather than discrete ones is also worth considering for future research.

Our regression analysis identifies the C9orf72 hexanucleotide repeat expansion as the only mutation in the Answer ALS cohort that independently accelerates the data-driven estimate of disease progression speed, consistent with prior reports of shortened survival in C9orf72-associated ALS^39,40^. Mechanistically, our GSEA of patient-derived iPSC-MNs revealed a coherent molecular signature implicating global translational inhibition at the center of fast progression. Meanwhile, SOD1 inclusions formed in patients with SOD1 mutations are independent of TDP-43^53^ and may not directly involve the translational machinery. The prognosis of SOD1 mutation patients varies depending on the mutation type, with D90A and H46R showing relatively mild clinical courses^54,55^, while the structurally unstable A4V mutation causes a severe ALS progression^56^. Due to the lack of information on mutation types, further investigation is necessary; however, the results from this study suggested that the progression speeds in SOD1 mutation patients were relatively slow, and direct ribosomal interference, which is present in C9orf72 and TDP-43 proteinopathies but rarely observed in most SOD1 variants, may accelerate disease progression. Additionally, proteomics analysis of iPSC-MNs revealed a tendency for proteins with negative effects on disease progression speed to outnumber those with positive effects. That is, lower expression levels of many proteins were associated with faster disease progression (Fig. 5a). The converging results of this study support a unified concept: the capacity of motor neurons to maintain protein synthesis balance under aggregation stress is a major determinant of ALS progression speed.

We evaluated biological covariates in a post-hoc manner to avoid imposing a specific parametric form for covariate effects within the latent progression model during model fitting. An important interpretational caveat is whether this “speed signature” reflects a predetermined trait or a secondary consequence of disease. We emphasize that the “speed” parameter is not assumed to be purely genetic; it can reflect a mixture of modifiers. Our genetic and iPSC-derived motor-neuron omics analyses are intended to probe the donor-linked component of the speed parameter in a hypothesis-generating manner. Notably, the possibility that the observed signatures reflect secondary disease-related programs cannot be excluded, and experimental validation will be required.

A limitation of our biological association analyses is the modest sample size of subjects with available genetic and proteomic measurements in the cohorts. This constrains statistical power, particularly for detecting modest effects after correction for multiple testing. We accordingly present these analyses as exploratory and primarily intended to generate hypotheses for future studies in larger multi-omics cohorts.

## Methods

### Cohorts and data pre-processing

This study used secondary human participant data obtained from the Answer ALS cohort and the PRO-ACT Database; these data had been de-identified or anonymized prior to access. The secondary analysis was reviewed and approved by the Nagoya University Institutional Review Board (IRB; approval number: 2025-0461). The original Answer ALS study and the clinical trials contributing data to PRO-ACT were conducted under the relevant institutional approvals and informed consent procedures. The present analysis was performed in accordance with the applicable data-use agreements and database terms and conditions. The Answer ALS cohort^35^ was used to estimate disease progression states and clusters, while the larger PRO-ACT^36^ cohort was used to validate the estimated states and clusters. The Answer ALS cohort includes information on the presence or absence of ALS gene mutations in patients and omics data from iPSC-MNs derived from patients, which were utilized in the analysis. In this study, we focused exclusively on patients diagnosed with ALS and classified as Limb-onset type. In both cohorts, patients were included if they had four or more hospital visits, had ALSFRS-R scores available, and had a total ALSFRS-R score of 21 or higher at the start of follow-up. Because we required four or more ALSFRS-R visits, patients with very rapid progression or early dropout may be under-represented, potentially introducing selection bias. The PRO-ACT cohort includes patients with very long follow-up periods and a large number of visits, so we set the maximum number of follow-up visits to 20 and the maximum follow-up period to 200 weeks, and only patients meeting these criteria were included in the analysis. For the PRO-ACT cohort, we excluded subjects with more than 20 visits or follow-up longer than 200 weeks. These subjects were removed entirely from the analysis rather than administratively truncated, to reduce the disproportionate influence of unusually dense or long follow-up records and to harmonize the effective observation window across participants. Patients with very slow progression may be under-represented, potentially introducing selection bias. For AnswerALS cohort, among 585 patients with limb-onset ALS, a total of 264 patients met these criteria and are included in the analysis, while 321 patients are excluded. For PRO-ACT cohort, among 7466 patients with limb-onset ALS, 2565 patients are included, while 4901 patients are excluded. Basic characteristic values for each cohort are shown in Supplementary Table 1.

For the ALSFRS-R score, the value recorded for questions 5a and 5b was used as the score for question 5. In addition, patients evaluated by ALSFRS-R, not by ALSFRS were included in the analysis. When any one of the scores for each question was missing, it was assumed that there was no observation point. That is, each observation point was ensured to have exactly 12 scores.

The Answer ALS cohort data contain information about major ALS-associated gene variants. The presence of the mutations in patients was detected based on whole-genome sequencing (WGS) results, or through the record in “ClinReport Mutations Details,” or, for mutations involving expansion of repetitive sequences, through records of the number of repeat sequences. The repeat expansion mutations were defined as being present when they met the following criterion^57,58^:

The number of repeats in C9orf72 $\ge 100$

The number of repeats in ATXN2 $\ge 27$

### IPS-CT-HMM: individual-progression-speed continuous-time hidden Markov model

The ALS pathology’s progression and clinical observation are modeled with an individual-progression-speed continuous-time hidden Markov model (IPS-CT-HMM). Our IPS-CT-HMM extends a standard CT-HMM by introducing a scalar patient-specific speed parameter that rescales the transition rates. We assume that a patient ${i}(=1,\,\ldots ,{I})$ has ${n}(=\mathrm{1,2},\ldots ,{N}_{i})$-th observation at times ${\left\{{t}_{i,n}\right\}}_{n=1}^{{N}_{i}}.\,$The time interval of the observation of patient *i* is hence,

$${\Delta }_{i,n}={t}_{i,n+1}-{t}_{i,n}$$

The clinical data observed from patient $i$ at the $n$-th observation is denoted by ${X}_{i,n},$ an $M$-dimensional vector (in our application, $M=12$ ALSFRS-R items). The $m$-th component is denoted by ${x}_{i,n,m}$, which represents the sub-score of an ALSFRS-R item and is a categorical variable ${x}_{i,n,m}\in \{0,\,1,\,2,\,3,\,4\}$. The latent disease progression state of ALS progression at the $n$-th observation is denoted by ${s}_{i,n}\in \{1,\,2,\,\ldots ,{K}\}$. Each sub-score ${x}_{i,n,m}$ is probabilistically generated depending on the latent disease state ${s}_{i,n}$ according to a categorical emission probability distribution parametrized by $\varPhi$:

$$p({x}_{i,n,m}=l|{s}_{i,n}=j;\varPhi )={\varPhi }_{j,m,l}$$

In this formulation, the observed score is not assumed to be constant within a latent state. Rather, each latent state is characterized by an item-specific categorical distribution over the possible score values, allowing multiple observed values to occur within the same state. Conditional on the current latent state, the current observed score does not directly depend on the previous observed score; instead, temporal dependence is mediated through the latent-state dynamics governed by the continuous-time transition process.

The probability of transition from state $j$ at time ${t}_{i,n}$ to state $k$ at time ${t}_{i,n+1}$ is defined with the matrix exponential as

$$p({s}_{i,n+1}=k|{s}_{i,n}=j)=P{\left({\Delta }_{i,n}\right)}_{j,k}$$

$$P\left({\Delta }_{i,n}\right)={e}^{{\exp (v}_{i}){\Delta }_{i,n}Q}$$

where $Q$ is a state transition rate matrix and ${v}_{i}$ denotes a scalar patient-specific progression-speed parameter, whose exponential scales the transition frequency for that patient. We assumed that ${v}_{i}$ follows a normal distribution with a mean of zero.

$${v}_{i}{\mathscr{\sim }}{\mathscr{N}}(0,\,{\sigma }_{v}^{2})$$

We set the standard deviation of progression speed to a broad value (${\sigma }_{v}=5$, unless otherwise noted) to avoid excessive shrinkage of individual speeds. (Sensitivity analyses for ${\sigma }_{v}\,$ are provided in Supplementary Table 4). A standard CT-HMM is recovered as a special case when the speed parameter is fixed to a constant across patients (e.g., ${v}_{i}=0$ for all). The diagonal elements of $Q$ are defined as ${q}_{j,j}=-{\sum }_{k\ne j}{q}_{j,k}$, which means that the summation of elements of $Q$ in each row equals zero. In neurodegenerative diseases, it is currently impossible for disease progression to be reversed, i.e., for the loss of nerve cells to be improved. Therefore, we assumed that the progression state would not return to its original state. In other words, the state transition rate matrix was set as an upper triangular matrix. We did not remove transient improvements observed in ALSFRS-R scores; because backward transitions are prohibited, such increases are treated as observation-level fluctuations captured by the probabilistic model rather than recovery of latent disease state. In addition, since only patients who did not show a significant decline in ALSFRS-R at the start of follow-up were included (total ALSFRS-R $\ge 21$), we restricted the initial latent state at the first observation to the early-stage states ($j=1,\,2,\,\ldots ,\,\left\lfloor /2\right\rfloor$), preventing implausible use of late-stage states at the start.

Derivation of the EM algorithm of CT-HMM was described in the past literature^20,59^. In an IPS-CT-HMM case, the complete log-likelihood is formulated as follows:

$$\log L=\,\mathop{\sum }\limits_{i=1}^{I}\left\{\mathop{\sum }\limits_{j=1}^{K}\mathop{\sum }\limits_{k\ne j}^{K}\left\{\log ({{e}^{{v}_{i}}q}_{j,k}){\eta }_{i,j,k}+{{e}^{{v}_{i}}q}_{j,j}{\tau }_{i,j}\right\}+\mathop{\sum }\limits_{n=1}^{{N}_{i}}\mathop{\sum }\limits_{m=1}^{M}\log p\left({x}_{i,n,m}\right){s}_{i,n};{\varPhi }_{{S}_{i,n},m})-{v}_{i}^{2}/2{\sigma }_{v}^{2}\right\}+{const}.,$$

where ${\eta }_{i,j,k}$ is the times of individual $i$‘s transition from state $j$ to state $k$, and ${\tau }_{i,j}$ is the individual $i$‘s staying times at state $j$. Thus, the expected complete log-likelihood is as follows:

$${\mathscr{Q}}=\mathop{\sum }\limits_{i=1}^{I}\left\{\mathop{\sum }\limits_{j=1}^{K}\mathop{\sum }\limits_{k\ne j}^{K}\left\{\log ({{e}^{{v}_{i}}q}_{j,k}){\mathbb{E}}\left[{\eta }_{i,j,k}|{X}_{i},{T}_{i},Q,{v}_{i}\right]\right\}+\left\{{{e}^{{v}_{i}}q}_{j,j}{\mathbb{E}}\left[{\tau }_{i,j}|{X}_{i},{T}_{i},Q,{v}_{i}\right]\right\}+\mathop{\sum }\limits_{n=1}^{{N}_{i}}{\mathbb{E}}\left[\left(\log p\left({x}_{i,n}\right){s}_{i,n}\right)|{X}_{i},\,{T}_{i},\,Q,\,{v}_{i},\varPhi \right]-{v}_{i}^{2}/2{\sigma }_{v}^{2}\right\}+{const}.,$$

where the calculation methods for the expectation of ${\eta }_{i,j,k}$ and ${\tau }_{i,j}\,$ were based on the methods described in the original paper on the CT-HMM algorithm^20^ (E-step).

In the M-step, the parameters were updated according to the following equations, which are the partial derivative of the expected complete log-likelihood:

$${\pi }_{j}^{\mathrm{new}}=\left\{\begin{array}{l}\frac{{\sum }_{i=1}^{I}{\gamma }_{i,\,1,\,j}}{{\sum }_{i=1}^{I}{\sum }_{k=1}^{\left\lfloor K/2\right\rfloor }{\gamma }_{i,1,j}},\,\left(j=1,\,2,\,\ldots \left\lfloor \frac{K}{2}\right\rfloor \right)\\ \,0,\,\,\,\,\,\,\,\,\,\,\,\,\,\,\,\,\,\,\,\,\,\,\,\left(j=\left\lfloor \frac{K}{2}\right\rfloor +1,\,\ldots ,K\right)\end{array}\right.\,$$

$${q}_{j,k}^{\mathrm{new}}=\left\{\begin{array}{l}\frac{{\sum }_{i=1}^{I}{\mathbb{E}}\left[{\eta }_{i,j,k}|{X}_{i},{T}_{i},Q,{v}_{i}\right]}{{\sum }_{i=1}^{I}{e}^{{v}_{i}}{\mathbb{E}}\left[{\tau }_{i,j}|{X}_{i},{T}_{i},Q,{v}_{i}\right]},\,\left(j\ne k\right)\\ -{\sum }_{j\ne k}{q}_{j,k}^{\mathrm{new}},\,\,\,\,\,\,\,\,\,\,\,\,\,\,\,\,\,\,\,\,\,\left(j=k\right)\end{array}\right.$$

$${\varPhi }_{j,m,l}^{\mathrm{new}}=\frac{{\sum }_{i=1}^{I}{\sum }_{n=1}^{{N}_{i}}\,{\gamma }_{i,n,j}{\bf{1}}[{x}_{i,n,m}=l]}{{\sum }_{i=1}^{I}{\sum }_{n=1}^{{N}_{i}}\,{\gamma }_{i,n,j}}$$

where $\pi$ is the initial state probability and ${\gamma }_{i,n,j}$ is the posterior probability that patient $i$ is in latent state ${j}$ at observation $n$, given the entire observation sequence ${X}_{i}$ and visit times ${T}_{i}$. ${v}_{i}^{{\rm{new}}}$ can be obtained by numerically solving the following equation. In this study, we used the BFGS algorithm to calculate the solution:

$$\frac{\partial }{\partial {v}_{i}}{\mathscr{Q}}{\mathscr{(}}{v}_{i})={\sum }_{j=1}^{K}{\sum }_{k\ne j}^{K}{\mathbb{E}}\left[{\eta }_{i,j,k}|{X}_{i},{T}_{i},Q,{v}_{i}\right]\,+{e}^{{v}_{i}}{\sum }_{j=1}^{K}{\sum }_{k\ne j}^{K}{q}_{j,j}{\mathbb{E}}\left[{\tau }_{i,j}|{X}_{i},{T}_{i},Q,{v}_{i}\right]-{v}_{i}/{\sigma }_{v}^{2}=0$$

For BFGS optimization at each M-step, default convergence criteria of SciPy module were used, requiring the gradient norm to be below $1\times {10}^{-5}$.

The E-step and M-step were iterated for a maximum of 20 iterations, with early stopping when the relative improvement in the observed-data log-likelihood fell below 0.1%. After parameter estimation, the patient-specific sequence of disease progression states for each patient ${\left\{{s}_{i,n}\right\}}_{n=1}^{{N}_{i}}\,$ were decoded using Viterbi algorithm, which is a deterministic dynamic-programming step. The number of the latent disease states were determined to maximize held-out likelihood via 10-fold patient-level cross-validation^5^. We selected this cross-validation–based approach because parameter counting in our model is non-trivial due to generator constraints and subject-specific speed parameters, making information-criterion approaches less straightforward to apply.

### Clustering of the trajectories of the disease progression states

By clustering the estimated trajectories of disease progression states, we obtained clusters of patients showing similar disease progression pathways. Here, “progression pathway” refers to the realized sequence of latent disease states under the shared probabilistic transition model; the individual speed parameter time-scales transitions but does not determine which transition branch is realized. First, the estimated state series included information on the time when observations corresponding to the estimated states were made, but since we focused only on the pathways, we excluded time-related information. We also removed duplicate states from the state series and obtained a unique set of states for each patient. Next, we defined the distance between states using emission probability distributions. Specifically, we defined the Wasserstein distance between the emission probability distribution of each score in a given state and the emission probability distribution in another state as the distance between those states.

$$d\left(j,k\right)={W}_{1}({\Phi }_{j,-,-},{\Phi }_{k,-,-})$$

Using this definition of distance between states, we calculated the dissimilarity between sequences as Dynamic Time Warping (DTW) cost and defined the similarity of sequences as the negative value of DTW cost. Then, we performed hierarchical clustering using the Ward’s method to obtain patient clusters. The number of clusters was set to be sufficient for identifying clusters with different state transition branching in the estimated transition rate matrix.

### Estimation for the PRO-ACT cohort patients using the results from the Answer ALS cohort

The parameters common for all patients, which include the state transition rate matrix, the probability of each sub-score occurring in each state, and the initial state probability, were provided to the IPS-CT-HMM as known values, estimated from the AnswerALS patient data. Then, PRO-ACT cohort patient data was provided to estimate the disease progression speed and the trajectory of disease progression states specific to each PRO-ACT cohort patient.

Cluster assignment for PRO-ACT cohort patients was performed based on disease state trajectories, where clusters derived from AnswerALS cohort patient data were assigned. The disease state trajectories estimated for PRO-ACT cohort patients were compared to those of AnswerALS cohort patients. For each combination, the DTW cost was calculated as was done during the AnswerALS clustering. Subsequently, the AnswerALS patient cluster exhibiting the closest disease state transition was assigned as the disease progression cluster for each PRO-ACT cohort patient.

### Survival and functional milestone analyses

We evaluated the clinical utility of estimated progression speed and pathway (cluster) using time-to-event analyses. Time was measured in weeks from the first ALSFRS-R assessment. For overall survival, event status/timing was obtained from mortality information; individuals without a recorded event were censored at their last available clinical observation.

To define functional milestone endpoints, we computed ALSFRS-R domain subtotals: Q1–Q3, Q4–Q6, Q7–Q9, and Q10–Q12. For each domain, we evaluated time to reach a severe impairment threshold (domain subtotal ≤6). The event time was defined as the first visit at which the domain subtotal met or fell below the threshold; subjects not reaching the threshold were censored at their last ALSFRS-R visit.

We generated Kaplan–Meier curves stratified by speed tertiles and by pathway cluster and assessed differences using log-rank tests. We fit Cox proportional-hazards models with progression speed standardized (z-score) so that hazard ratios represent a 1 SD (standard deviation) increase in speed. Cluster was included as a categorical variable via dummy indicators. Multivariable Cox models additionally included baseline clinical covariates (sex, age at symptom onset) to evaluate whether pathways provide prognostic information beyond speed and baseline features.

### Comparison with CT-HMM in PRO-ACT trajectory prediction

To quantify the predictive benefit of patient-specific progression speed, we performed a forecasting analysis in PRO-ACT comparing the IPS-CT-HMM with a non-personalized uniform-speed CT-HMM. We trained the two models separately on the AnswerALS cohort and transferred the corresponding patient-common parameters to PRO-ACT for each model. For each PRO-ACT subject, visits were ordered by time from the first ALSFRS-R assessment. The first three ALSFRS-R observations were used as training data, and all subsequent observations occurring within 52 weeks (one year) after the third observation were used as prediction targets. We restricted this analysis to patients with baseline total ALSFRS-R ≥ 36 at the first observation (*n* = 1798) because the score exhibits pronounced floor effects and the restriction provides a more reliable setting to evaluate prediction performance. Predictions were computed using soft state posteriors at the third observation and continuous-time propagation of the latent-state distribution. Specifically, we computed the posterior state distribution ${\gamma }_{{\rm{i}},3}=p({s}_{i,3}| {x}_{i,1:3})$ using the forward–backward algorithm, and propagated it to each future target observation ${n}_{* }$ via $p({s}_{i,{n}_{* }})={\gamma }_{i,3}\exp (\exp \left({v}_{i}\right){\Delta }_{i,* }Q)$, where ${\Delta }_{i,* }={t}_{i,{n}_{* }}-{t}_{i,3}$. In the IPS-CT-HMM, a subject-specific progression speed parameter ${v}_{i}$ was estimated from the first three observations and used to compute latent state distribution. In the uniform-speed CT-HMM, ${v}_{i}$ was fixed to 0 for all subjects. For the estimation, the standard deviation of progression speed was set as ${\sigma }_{v}=3$ considering the small number of observation points. Performance was evaluated using point-prediction error for total ALSFRS-R (MAE, RMSE, and ${R}^{2}$) computed from the expected score under the predictive distribution, and probabilistic forecasting performance using predictive log-likelihood of future observations under the model.

### Association analysis of genetic mutations with estimated disease progression speed

We modeled estimated progression speed as the dependent variable using ordinary least squares (OLS) regression implemented in Python. The primary explanatory variable was the presence of the genetic mutation of interest (no = 0, yes = 1). Covariates were sex (female = 0, male = 1), age at symptom onset (normalized to have a mean of 0 and a variance of 1) and history of riluzole use (no = 0, yes = 1). The model was ${v}_{i}={\beta }_{0}+{\beta }_{1}{\left\{{\rm{Existence\; of\; the\; Mutation}}\right\}}_{i}+{\beta }_{2}{\left\{{\rm{Sex}}\right\}}_{i}+{\beta }_{3}{\left\{{\rm{Age\; at\; symptom\; onset}}\right\}}_{i}+{\beta }_{4}{\left\{{\rm{Riluzole\; history}}\right\}}_{i}+{\epsilon }_{i}$

With ${\epsilon }_{i}$ assumed i.i.d. with mean zero. Patients with any missing values in model variables were excluded. Our primary parameter of interest was ${\beta }_{1}$, the coefficient on the mutation indicator. Two-sided t-tests at $\alpha$= 0.05 were used for inference; effects were deemed statistically significant when the two-sided 95% confidence interval for ${\beta }_{1}$ excluded zero. A positive ${\beta }_{1}$ indicates higher estimated progression speed in carriers relative to non-carriers. Model assumptions were evaluated by visual inspection of residual-versus-fitted plots and normal Q–Q plots to assess linearity, homoscedasticity, and approximate normality of residuals.

We additionally assessed the continuous association between estimated progression speed and C9orf72 repeat expansion length by computing Pearson’s correlation between progression speed and log10-transformed repeat length among participants with available repeat-length measurements. We also fitted an OLS model in which the binary mutation indicator was replaced by log10 repeat length while retaining the same covariates.

### Discovery of biological association of the disease progression speed

We obtained transcriptome and proteome data obtained from motor neurons differentiated from patient iPS cells from the AnswerALS database. First, we perform multivariable regression, where estimated progression speed as the dependent variable and the expression of a gene/protein as the main explanatory variable with covariates including sex, age of symptom onset, riluzole exposure status and the existence of C9orf72 mutation. Next, we ordered genes/proteins based on their $-{\log }_{10}({\rm{p}}-{\rm{value}})$ from the multivariable regression results and performed gene enrichment analysis (GSEA). Here, the order was arranged by dividing the genes/proteins into those with positive effects on expression levels and those with negative effects on estimated progression speed, based on the multivariable regression results. Genes/proteins with positive effects were sorted in descending order by $-{\log }_{10}(p-\mathrm{value})$. Subsequently, genes/proteins with negative effects were sorted in ascending order by $-{\log }_{10}({\rm{p}}-{\rm{value}})$. GSEA was performed using pyGSEA. The gene sets used in the GSEA were “KEGG_2019_Human”, “MSigDB_Hallmark_2020”, “GO_Biological_Process_2018”, ‘GO_Molecular_Function_2018’, and “GO_Cellular_Component_2018”. The minimum and maximum size of the elements in the gene set was set as 15 and 1000, respectively, and the number of permutation times was set as 1000 times.

### Prediction of the disease progression speeds and progression clusters

We evaluated whether the disease progression speed and progression cluster of patients could be predicted from clinical information at the start of tracking. The AnswerALS dataset was separated into a training dataset that was used to train machine learning models and test data that was used to evaluate the performance of the trained machine learning models. The test dataset contained data of patients who do not belong to the training dataset. Cross-validation was performed in the leave-one-out manner. For the estimation in test data, the standard deviation of progression speed was set as ${\sigma }_{v}=3$ considering only a patients included in the estimation. The assignment of clusters to patients within each cross-validation set was performed by defining characteristic residence states for clusters derived from the full AnswerALS cohort data. We estimated the state trajectories of patients within each cross-validation set and assigned the cluster whose characteristic staying state most closely matched their estimated disease state trajectory.

For the predictors, we used sex, age at symptom onset, the presence of the genetic mutations (C9orf72 and SOD1) and the sum of the scores for each of the four domains of the ALSFRS-R. When the analysis was configured to include omics features, the principal components of gene/protein expression levels were added to the pool of predictors.

To predict the estimated progression speed from the constructed predictors, we fitted an L2-regularized linear model (a Ridge regressor). the regularization strength to minimize mean squared error was selected by 5-fold cross-validation within the training set using a grid. The best model was refit on the full training set and used to generate predictions for the held-out test set.

We also predicted the categorical cluster label using a linear-kernel support vector machine. To capture the joint information in clinical features and disease speed, the classifier’s input comprised the same predictor variables used for predicting progression speeds plus a single additional feature: the progression speed estimated from whole observations for training and the predicted progression speed from the Ridge regressor for test. The regularization parameter $C$ was tuned by 5-fold cross-validation over $C\in \{0.01,\,0.1,\,1,\,10,\,100\}$ with accuracy as the scoring metric, where class imbalance was adjusted. The selected SVM was then applied to the held-out test set to obtain predicted cluster labels.

### Benchmarking against an ENCALS-like Cox model

To benchmark DiSPAH against established prognostic tools used in ALS practice and clinical trials, we constructed an ENCALS-like^43^ baseline Cox proportional hazards model and compared it with extensions incorporating DiSPAH-derived features. We used the first ALSFRS-R observation (day0) as the baseline time origin. In Answer ALS, onset, diagnosis, and death timings are recorded as days relative to day 0; we therefore computed time-to-event outcomes and timing-derived predictors on this relative-day scale.

Model A (ENCALS-like) included seven predictors: age at onset, diagnostic delay, forced vital capacity (FVC), El Escorial diagnostic category, frontotemporal dementia (FTD), C9orf72 repeat expansion status, and a pre-baseline progression-rate proxy ΔFRS^60^, defined as

$$\Delta {FRS}=\frac{48-[{\rm{total\; ALSFRS}}-{\rm{R\; score\; at\; day}}0]}{[{\rm{months\; since\; onset\; day\; to\; day}}0]}$$

where months since symptom onset was computed from the relative onset timing. Because our cohort was restricted to limb-onset ALS, site of onset was excluded from the ENCALS predictor set^43^. We then evaluated incremental models incorporating predicted DiSPAH-derived features: predicted progression speed (continuous) and predicted pathway cluster (categorical, one-hot encoded with a reference category). For mortality prediction, the event was death and subjects were censored at the last ALSFRS-R observation. For milestone prediction, events were defined as the first observation where an ALSFRS-R domain sub-score fell to ≤6 (bulbar Q1–3, fine motor Q4–6, gross motor Q7–9, respiratory Q10–12), censoring at the last observation if the milestone was not reached. Discrimination was evaluated using patient-wise 5-fold cross-validated concordance index (C-index). Binary predictors with too few positive cases were removed during the training step to prevent overfitting; consequently, FTD and C9orf72 status were not retained.

## Supplementary information

Yada2025_DiSPAH_Supple_revised_unmarked

## Data Availability

Clinical data and iPSC-MN omics data from AnswerALS cohort are available for download from the Answer ALS data portal (https://dataportal.answerals.org). Clinical data from PRO-ACT cohort are available for download from the PRO-ACT database (https://ncri1.partners.org/ProACT/Home/Index).

## Code availability

All code used in the current study is available on the GitHub repository (https://github.com/yadapo/DiSPAH).
