# Supplementary material for: Decomposing heterogeneity in disease progression speeds and pathways
Source: NPJ Digit Med. 2026 May 12;9:562. doi: 10.1038/s41746-026-02665-8 (PMC13388994; doi:10.1038/s41746-026-02665-8)
Supplement: Supplementary file 1 — Yada2025_DiSPAH_Supple_revised_unmarked [file 41746_2026_2665_MOESM1_ESM.docx]

**Supplementary Information:**

**Decomposing heterogeneity in disease progression speeds and pathways**

Authors:

Yuichiro Yada^1,2*^, Honda Naoki^1,3,4*^

Affiliations:

1. Laboratory for Data-driven Biology, Nagoya University Graduate School of Medicine, 65, Tsurumai-cho, Showa-ku, Nagoya, Aichi, 466-8550, Japan
2. Institute for Advanced Research, Nagoya University, Furo-cho, Chikusa-ku, Nagoya, Aichi, 464-8601, Japan
3. Laboratory of Data-driven Biology, Graduate School of Integrated Sciences for Life, Hiroshima University, 1-3-1, Kagamiyama, Higashi-hiroshima, Hiroshima, 739-8526, Japan
4. Center for One Medicine Innovative Translational Research (COMIT), Nagoya University, 65, Tsurumai-cho, Showa-ku, Nagoya, Aichi, 466-8550, Japan

*Corresponding author: Yuichiro Yada

Address: Nagoya University Graduate School of Medicine, 65, Tsurumai-cho, Showa-ku, Nagoya, Aichi, 466-8550, Japan

Tel.: +81-52-744-1980

E-mail: [yada.yuichiro.k4@f.mail.nagoya-u.ac.jp](mailto:yada.yuichiro.k4@f.mail.nagoya-u.ac.jp)

*Co-Corresponding author: Honda Naoki

Address: Nagoya University Graduate School of Medicine, 65, Tsurumai-cho, Showa-ku, Nagoya, Aichi, 466-8550, Japan

Tel.: +81-52-744-1980

E-mail: [honda.naoki.t1@f.mail.nagoya-u.ac.jp](mailto:honda.naoki.t1@f.mail.nagoya-u.ac.jp)

**Supplementary Tables**

Supplementary Table 1: Basic characteristics of the cohort used in the present study

Only patients in each cohort who had records of visiting the hospital at least four times for ALSFRS-R assessments and whose onset location was recorded as limb-onset were included. Patients without recorded covariate values were excluded from the analyses of association with estimated progression speed and from the prognostic prediction. The values for ALSFRS-R Visit Times and Age At Symptom Onset represent the mean and standard deviation.

| Cohort | Number of patients extracted | ALSFRS-R Visit Times | Sex | Age At Symptom Onset | Riluzole Use History |
| --- | --- | --- | --- | --- | --- |
| AnswerALS | 264 | 5.78±1.72 | Female: 86  Male: 177  Not specified: 1 | 55.3±10.9 | TRUE: 177  FALSE: 87 |
| PRO-ACT | 2,565 | 8.88±3.58 | Female: 860  Male: 1705 | 53.7±11.2 | TRUE:1944  FALSE: 621 |

Supplementary Table 2: Characterization and occupancy of latent disease states learned by DiSPAH in AnswerALS.

Expected ALSFRS-R scores for each latent state were computed from the estimated emission probabilities as the expectation of each ALSFRS-R sub-score (0-4) and then summed to obtain the total score (0-48) and domain subtotals (bulbar, fine motor, gross motor, and respiratory; each 0-12). Higher scores indicate better function. Patients (n) and Patients (%) denote the number and percentage of patients who visited each state at least once based on Viterbi state sequences. Visits (n) and Visits (%) denote the number and percentage of clinical visits assigned to each state.

| **State** | **Expected Total** | **Expected Bulbar** | **Expected Fine motor** | **Expected Gross motor** | **Expected Respiratory** | **Patients (n)** | **Patients (%)** | **Visits (n)** | **Visits (%)** |
| --- | --- | --- | --- | --- | --- | --- | --- | --- | --- |
| 1 | 41.88 | 11.28 | 9.14 | 10.06 | 11.41 | 108 | 40.91 | 370 | 25.15 |
| 2 | 38.00 | 11.45 | 9.48 | 5.72 | 11.35 | 68 | 25.76 | 209 | 14.21 |
| 3 | 32.78 | 9.81 | 6.00 | 6.72 | 10.24 | 159 | 60.23 | 360 | 24.47 |
| 5 | 28.04 | 9.92 | 6.14 | 2.79 | 9.19 | 69 | 26.14 | 158 | 10.74 |
| 4 | 26.61 | 9.87 | 6.05 | 5.91 | 4.78 | 52 | 19.70 | 139 | 9.45 |
| 6 | 19.72 | 8.58 | 1.42 | 2.66 | 7.06 | 87 | 32.95 | 235 | 15.98 |

Supplementary Table 3: Reconstruction-based goodness-of-fit metrics

Reconstruction-based goodness-of-fit evaluation for ALSFRS-R in AnswerALS and PRO-ACT. At each observation, reconstructed scores were computed as the expected score under the learned emission distribution, averaged over posterior state probabilities (computed by the forward–backward algorithm). For each observation $n$of patient $i$, we reconstructed the expected sub-score as

$$\hat{x}_{i,n,m}= \sum_{j=1}^{K} \gamma_{i,n,j}\sum_{l=0}^{4} lp(x_{i,n,m}=l|s_{i,n}=j)$$

Reported are MAE and RMSE (points on the native score scale) and the coefficient of determination (R²). The total score is on the 0–48 scale; sub-scores are on the 0–4 scale.

|  | **AnswerALS** | | | **PRO-ACT** | | |
| --- | --- | --- | --- | --- | --- | --- |
| **Target** | **MAE** | **RMSE** | **R²** | **MAE** | **RMSE** | **R²** |
| Total score | 2.840 | 3.822 | 0.801 | 2.807 | 3.757 | 0.800 |
| Q1 | 0.650 | 0.857 | 0.194 | 0.619 | 0.834 | 0.269 |
| Q2 | 0.538 | 0.703 | 0.092 | 0.553 | 0.737 | 0.135 |
| Q3 | 0.581 | 0.774 | 0.188 | 0.544 | 0.752 | 0.260 |
| Q4 | 0.664 | 0.822 | 0.562 | 0.650 | 0.794 | 0.609 |
| Q5 | 0.625 | 0.770 | 0.639 | 0.621 | 0.769 | 0.668 |
| Q6 | 0.516 | 0.654 | 0.610 | 0.545 | 0.669 | 0.638 |
| Q7 | 0.597 | 0.748 | 0.586 | 0.588 | 0.744 | 0.627 |
| Q8 | 0.585 | 0.747 | 0.579 | 0.529 | 0.669 | 0.593 |
| Q9 | 0.659 | 0.928 | 0.564 | 0.595 | 0.826 | 0.609 |
| Q10 | 0.786 | 0.982 | 0.311 | 0.764 | 0.933 | 0.207 |
| Q11 | 0.766 | 1.078 | 0.406 | 0.579 | 0.807 | 0.187 |
| Q12 | 0.523 | 0.739 | 0.440 | 0.467 | 0.651 | 0.090 |

Supplementary Table 4: Sensitivity of estimated progression speed and clustering to the speed prior standard deviation $\sigma_{v}$. To assess whether the choice of $\sigma_{v}$ influences post-hoc analysis, we evaluated model fitting and trajectory clustering across $\sigma_{v}\in\{3,4,5,6,7\}$ and quantified stability relative to the baseline setting ($\sigma_{v}=5$). Reported are Pearson’s correlation of $v_{i}$ vs. the baseline ($\sigma_{v}=5$), Adjusted Rand Index (ARI) of cluster assignments vs. the baseline. Overall, estimated speeds were highly consistent in relative magnitude across settings and cluster assignments remained stable.

| $\boldsymbol{\sigma}_{\boldsymbol{v}}$ | **mean speed** | **speed std** | **speed Pearson’s correlation vs baseline** | **cluster ARI vs baseline** |
| --- | --- | --- | --- | --- |
| 3 | -0.194 | 1.504 | 0.961* | 0.678 |
| 4 | -0.305 | 1.706 | 0.992* | 0.894 |
| 5 | -0.401 | 1.882 | 1.000 | 1.000 |
| 6 | -0.467 | 1.975 | 0.993* | 0.902 |
| 7 | -0.531 | 2.074 | 0.992* | 0.912 |

*p-value<0.0001

**Supplementary Figures**


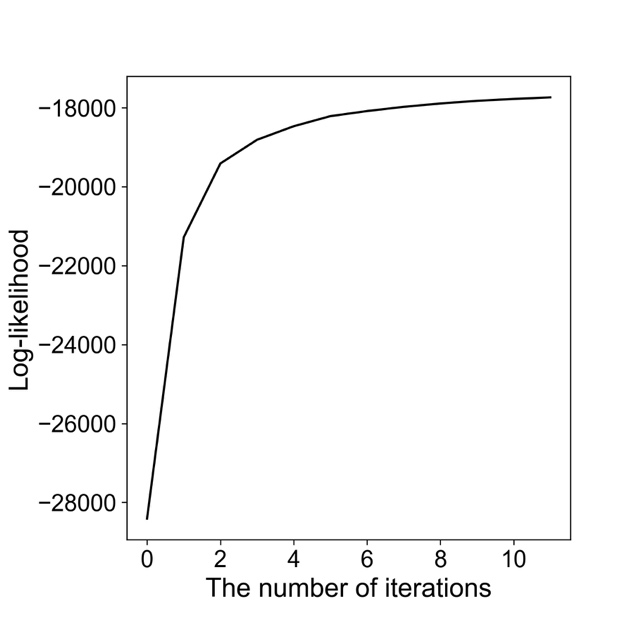


Supplementary Fig. 1: Convergence of model parameter update with EM algorithm

The model parameters of IPS-CT-HMM were updated using the EM algorithm to maximize the likelihood. The maximum number of updates was set to 20, and the updates were terminated when the increase in likelihood due to parameter updates fell below 0.1%.


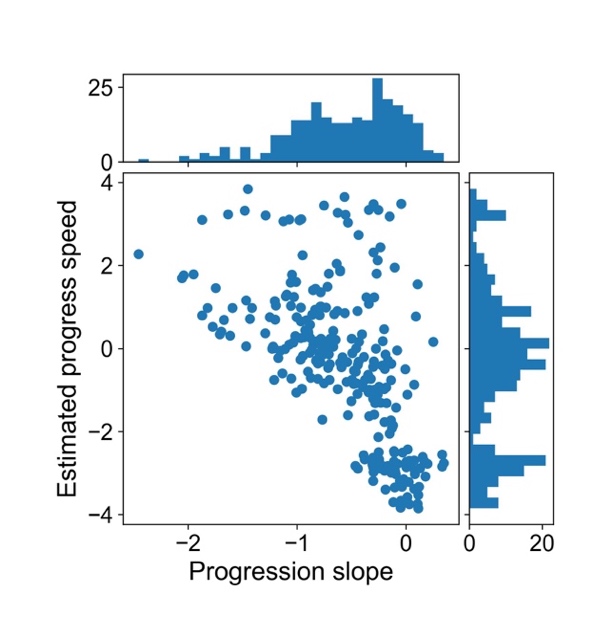


ALSFRS-R Progression slope

Supplementary Fig. 2: Relationship between the slope of the regression line for the ALSFRS-R total score and the estimated disease progression speed in the AnswerALS cohort
Scatter plot showing the estimated progression speed for patients with a given slope of the regression line for their ALSFRS-R total score. Among patients with a gentle slope, some are estimated to have a faster disease progression.


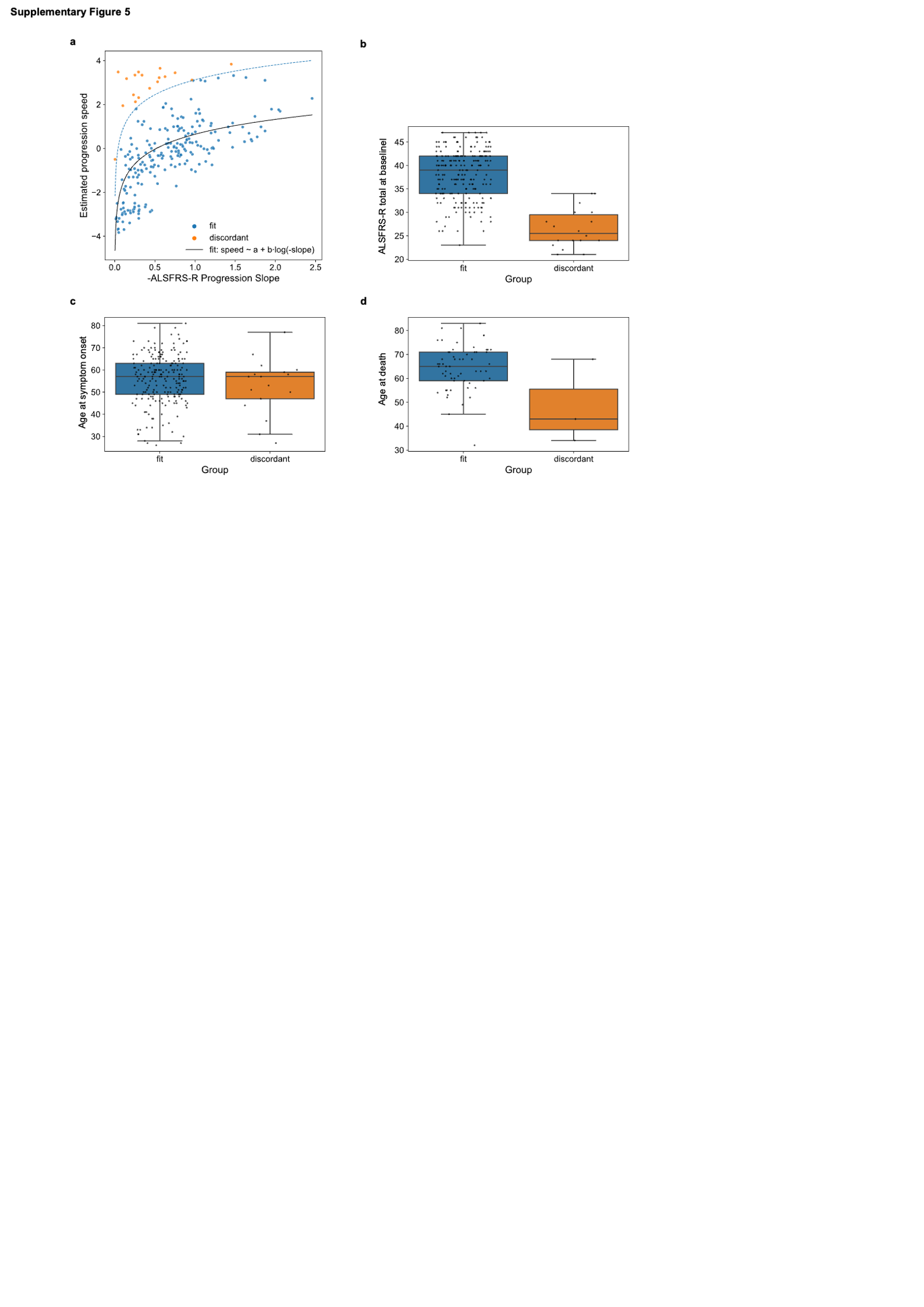


Supplementary Fig. 3: Clinical characterization of patients whose estimated progression speed and ALSFRS-R slope are discordant.
**a,** Relationship between the (negative) ALSFRS-R slope (x-axis; plotted as $-$ALSFRS-R Progression Slope) and the DiSPAH-estimated progression speed (y-axis). The black curve shows the fitted log relationship, $v_{i}\sim a+b\log(-\text{slope})$. Patients are classified as discordant (orange) when their estimated speed lies substantially above the fitted curve (one-sided criterion based on a robust residual threshold), and as fit (blue) otherwise.
**b–d,** Group comparisons between fit and discordant patients for baseline ALSFRS-R total score (**b**), age at symptom onset (**c**), and age at death (d; available only for a subset). Boxplots show median and interquartile range; whiskers indicate 1.5×IQR; dots represent individual patients.
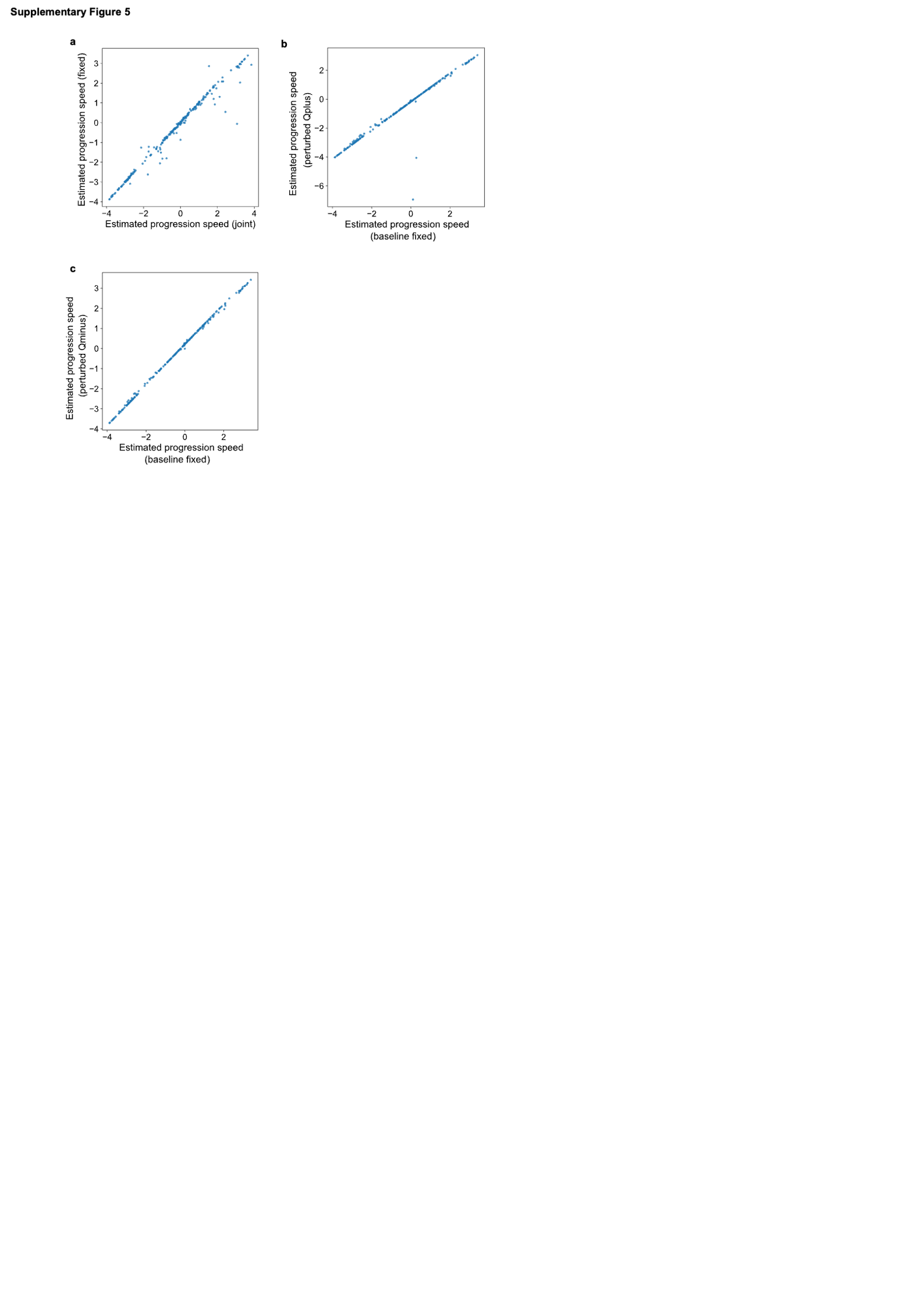


Supplementary Fig. 4: Robustness of relative progression speed estimates to fixing the transition-rate matrix.

**a,** Scatter plot comparing patient-specific speeds obtained under joint estimation of the transition rate matrix $Q$ and individual speeds (x-axis) versus speeds obtained under re-estimation of individual speeds while holding a pre-estimated transition-rate matrix fixed (y-axis). Each point represents one patient. Robustness was quantified by Pearson’s correlation ($r=0.992, p<0.0001$) indicating highly consistent relative speed ordering across estimation settings. **b,c,** Scatter plots comparing baseline fixed-$Q$ patient-specific progression speed estimates (x-axis) versus speed estimates obtained after perturbing the transition-rate matrix $Q$ and re-estimating patient speeds with the perturbed $Q$ fixed (y-axis). Off-diagonal transition rates were multiplied by $1\pm0.2$, and diagonal entries were recomputed to preserve the generator constraint (each row sums to zero). Relative speed estimates remained stable under both perturbations (Pearson’s correlation $r=0.962, p<0.0001$ for +20%; $r=0.999, p<0.0001$ for −20%). Each point represents one patient


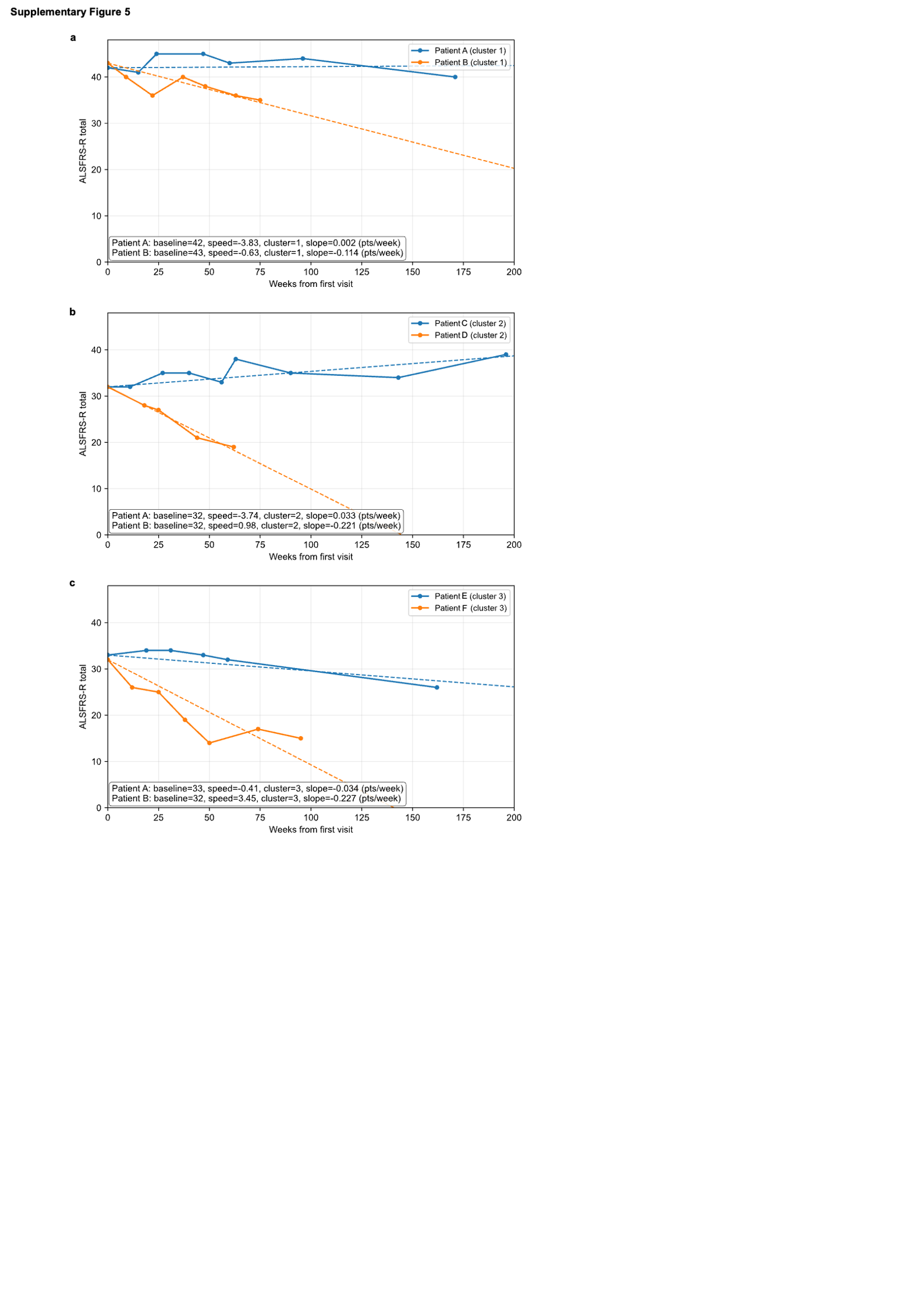


Supplementary Fig. 5: Clinical interpretation of estimated progression speed using illustrative patient pairs.
Example patient pairs selected from the Answer ALS cohort showing similar baseline total ALSFRS-R and the same cluster, but substantially different estimated progression speeds. For each pair, longitudinal total ALSFRS-R scores are plotted against weeks from the first visit. Faster-speed patients exhibit more rapid decline over subsequent visits, whereas slower-speed patients remain relatively stable over longer follow-up, illustrating how the inferred speed parameter captures clinically meaningful differences in disease tempo beyond baseline severity. Baseline total score, estimated speed, cluster assignment and slope of regression line for each patient are reported in the inset of each panel.

Supplementary Fig. 6: Detailed results of clustering the AnswerALS cohort data.

**a-f,** Figure showing the relationship between functional decline in domains among patients in each cluster identified in the AnswerALS cohort. Subtotals were calculated for each of the ALSFRS-R (Bulbar, Fine motor, Gross motor, Respiratory), and the subtotals for different domains at the same time point are shown.


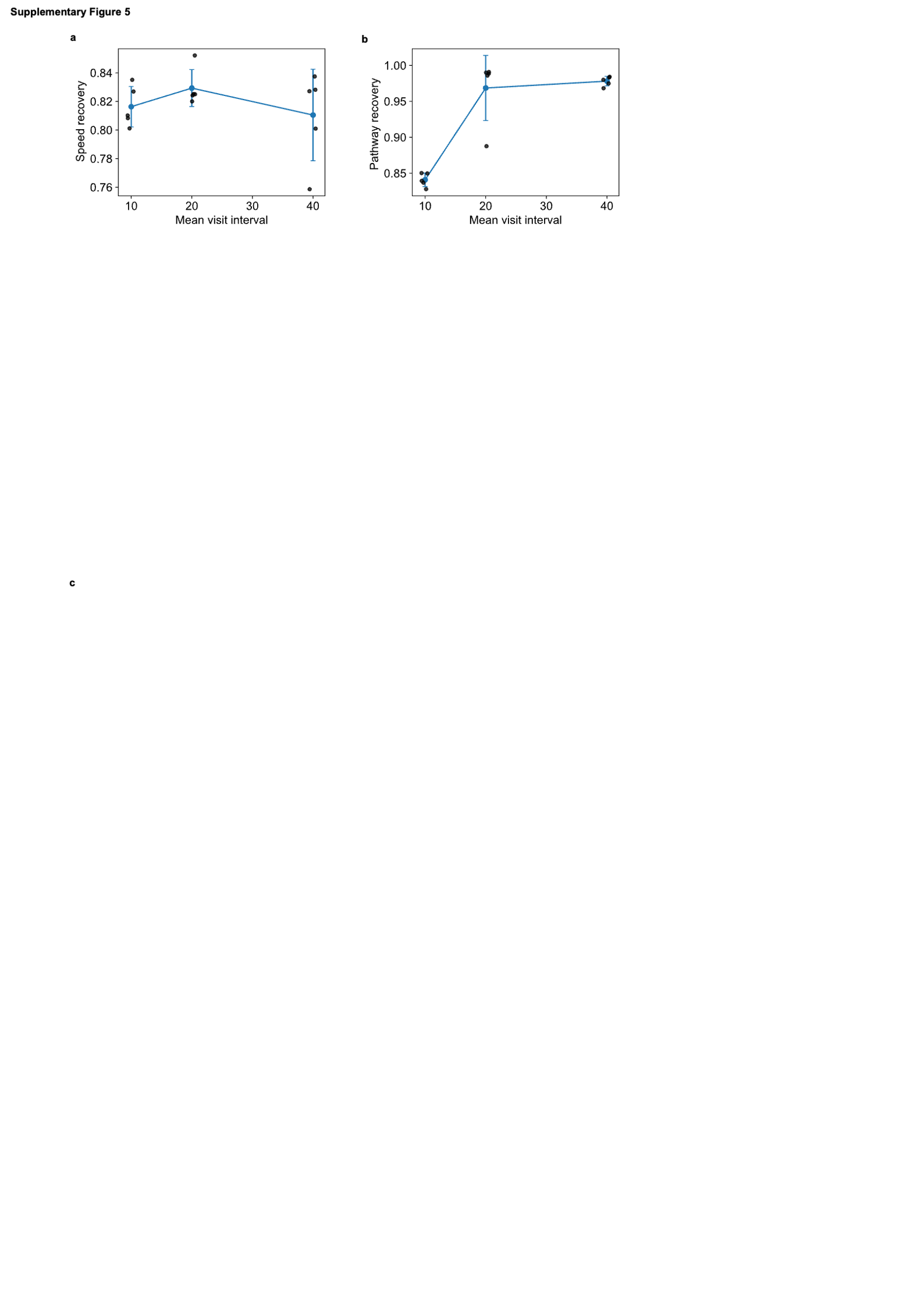


Supplementary Fig. 7: Simulation-based validation of patient-specific information estimation

**a,** Speed recoverability quantified as the Pearson’s correlation between true and estimated progression speeds. **b,** Pathway recoverability quantified as observation-point-wise agreement between true and estimated state sequences. Both are tested with three observation-schedule conditions with different mean visit intervals (10, 20, and 40 weeks).


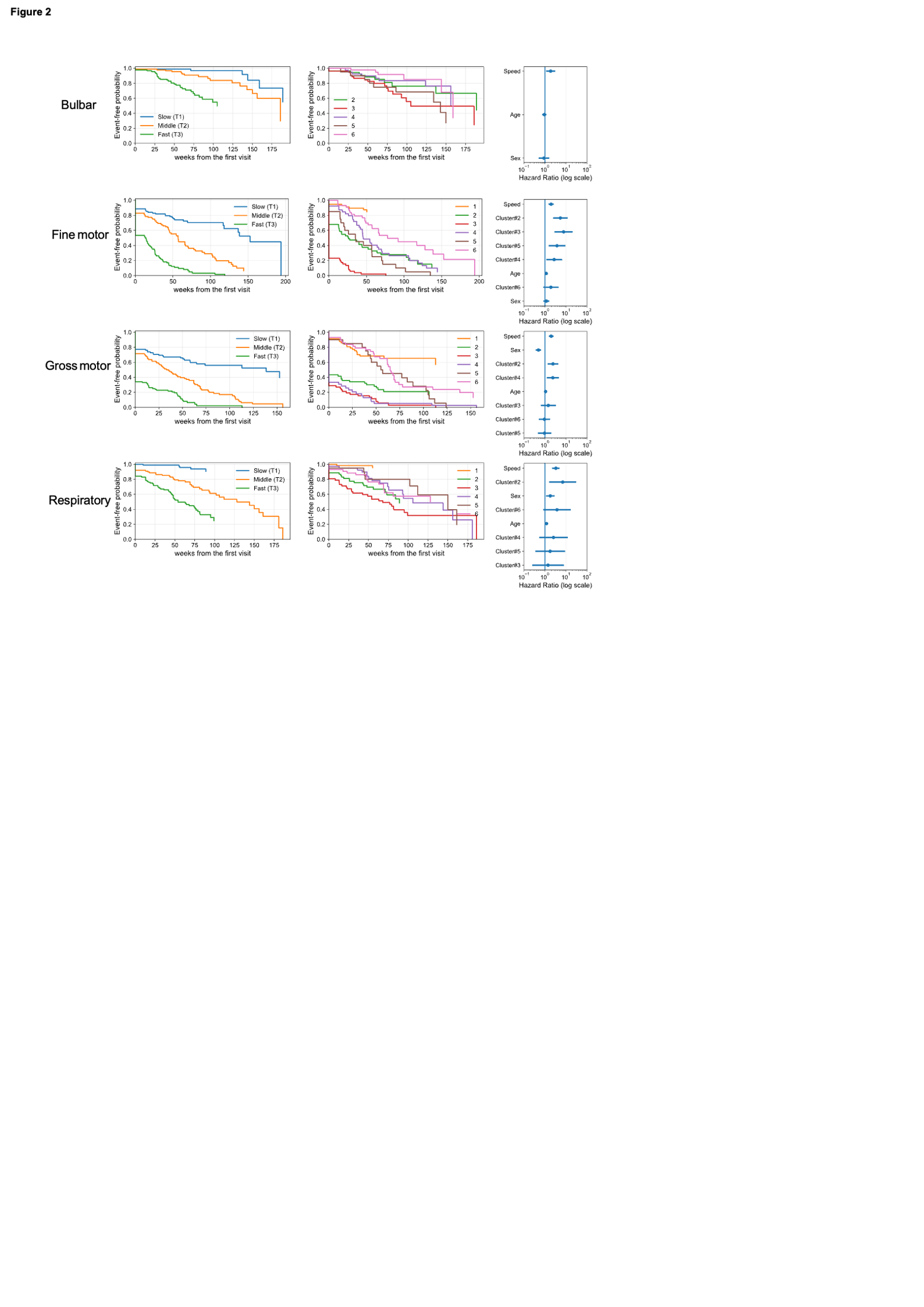


Supplementary Fig. 8: DiSPAH-derived progression speed and pathways stratify ALSFRS-R domain-specific functional milestone timing.

Kaplan–Meier curves for time to severe functional deterioration defined by ALSFRS-R domain subtotal ≤6 for each domain: (Bulbar) Q1–Q3, (Fine motor) Q4–Q6, (Gross motor) Q7–Q9, and (Respiratory) Q10–Q12. For each panel, curves are stratified by DiSPAH-derived speed tertiles and clusters. Time is measured in weeks from the first ALSFRS-R assessment. Event time is defined as the first visit at which the domain subtotal reaches ≤6; subjects not reaching the threshold are censored at their last ALSFRS-R visit. Right column shows multivariable Cox proportional-hazards model for each functional milestone including standardized progression speed, clusters and baseline clinical covariates (sex and age at symptom onset). Points denote hazard ratios (HR) and horizontal bars indicate 95% confidence intervals; the vertical line indicates HR = 1. Cluster #1 was used as the reference category. For the bulbar-domain milestone, pathway cluster indicators were omitted (due to insufficient events in some clusters).

Supplementary Fig. 9: Detailed results of clustering the PRO-ACT cohort data.

**a-f,** Figure showing the classification of PRO-ACT cohort patients and the relationship between the degree of decline in functional domains among patients in each cluster. For classification, each cluster identified in the AnswerALS cohort was referenced. Subtotals were calculated for each domain of the ALSFRS-R (bulbar, fine motor, gross motor, respiratory), and the subtotals for different domains at the same time point are shown.


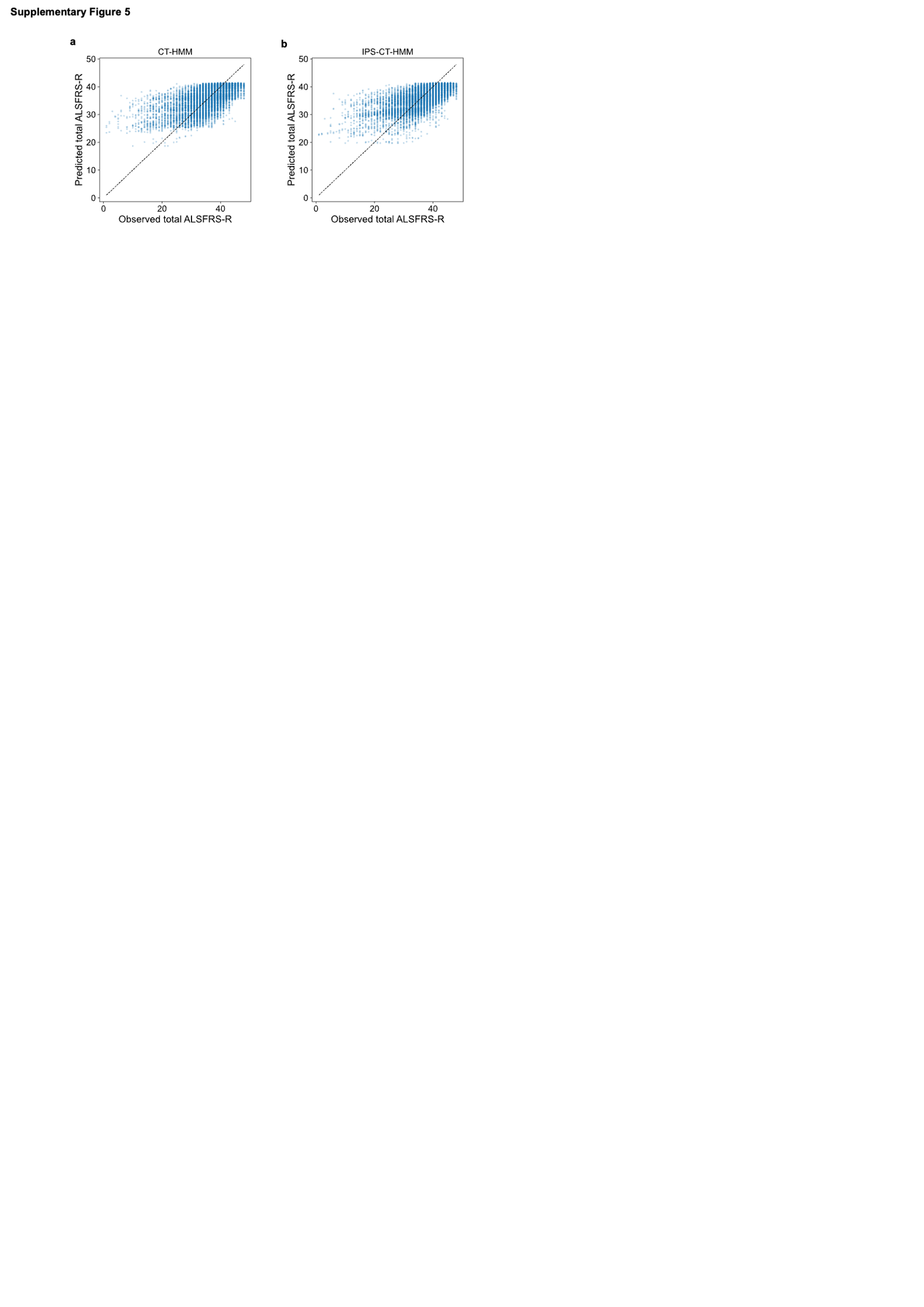


Supplementary Fig. 10: Comparison with CT-HMM in PRO-ACT trajectory prediction.
**a, b,** Scatter plots comparing observed and model-predicted total ALSFRS-R scores. Each point represents one held-out observation. The dashed diagonal line indicates perfect agreement ($y=x$). **a,** Uniform-speed CT-HMM (without individual speed scaling). **b,** IPS-CT-HMM (with an individual-specific progression-speed parameter). Both models were evaluated under the same prediction setting and data split, enabling a direct comparison of predictive accuracy attributable to the individualized speed component.


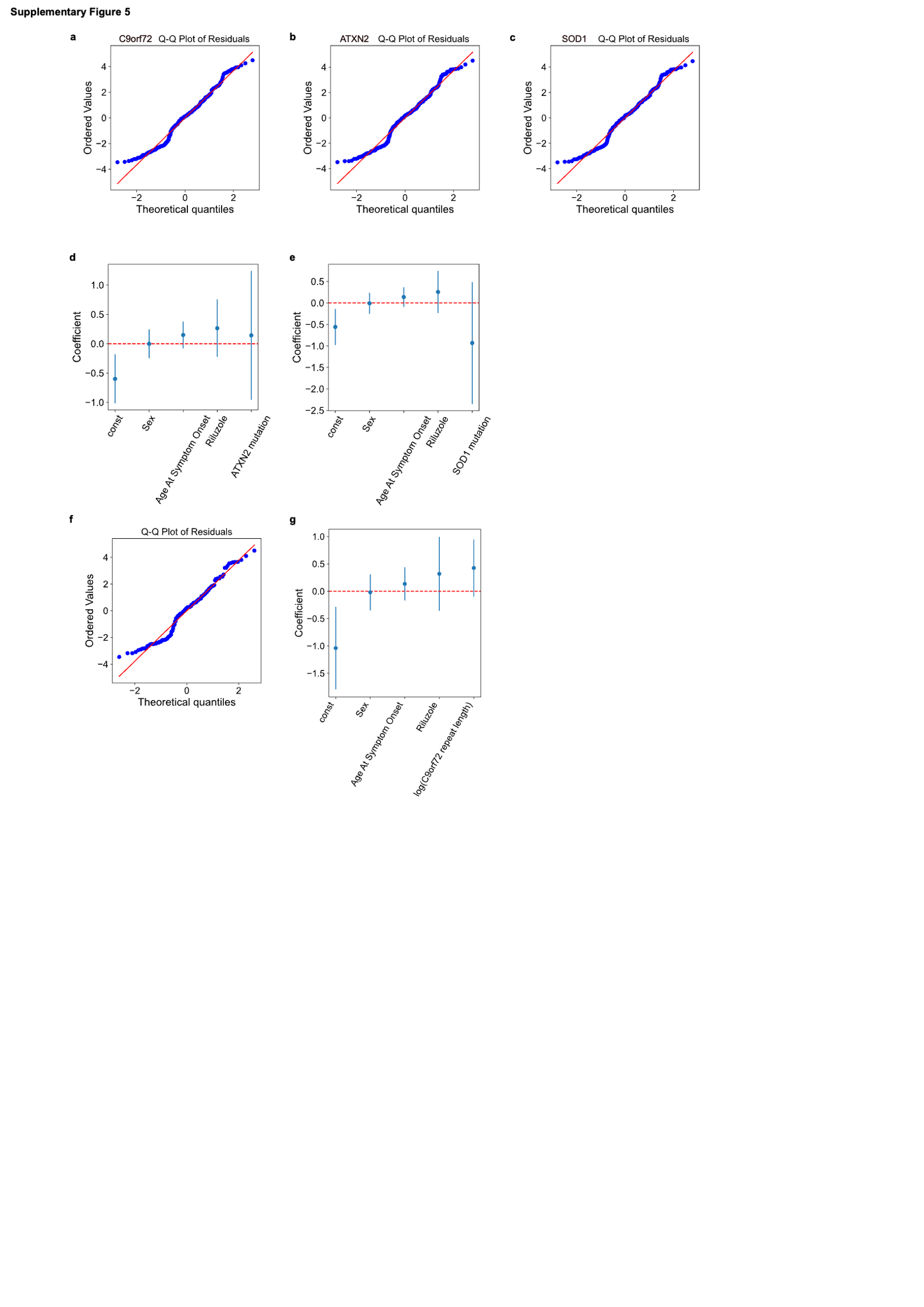


Supplementary Fig. 11: ALS-related genetic variants and C9orf72 repeat expansion length show no significant association with the estimated progression speed.

**a-c**, Q–Q plots of standardized residuals from multivariable regression with estimated progression speed as the target variable. The models include **a** C9orf72, **b** ATXN2, or **c** SOD1 genetic predictors. Blue circles show ordered residuals; the red line indicates theoretical normal quantiles. Approximate linearity supports the Gaussian error assumption. **d-e,** Point estimates (dots) and 95% confidence intervals (bars) for coefficients of the multivariable regression with estimated progression speed as the target variable. The models included the ATXN2 mutation term or **e** the SOD1 mutation term as a primary explanatory variable, adjusted for sex, age at symptom onset, and riluzole use. “const” denotes the model intercept and is not interpreted. **f–g,** Multivariable regression using log10-transformed C9orf72 repeat expansion length (among participants with repeat-length measurements) with the same covariates; **f** shows the Q–Q plot of standardized residuals and **g** shows coefficient estimates and 95% confidence intervals.

Supplementary Fig. 12: Comprehensive results of gene enrichment analysis on the association between gene/protein expression in patient-derived iPSCs-based motor neurons and estimated disease progression speed.

**a-b,** Extended examples of gene groups exhibiting significant fluctuations in relation to estimated progression speed, obtained from GSEA analysis in the transcriptome. **c**, Those obtained from the proteome.


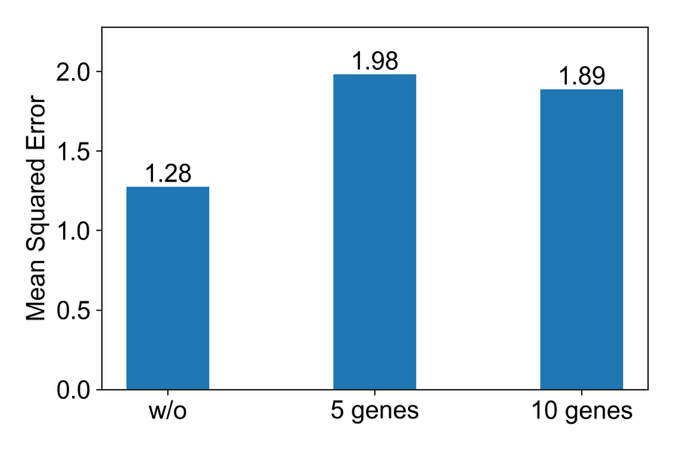


Supplementary Fig. 13: Prediction using transcriptome data obtained from the patient motor neurons in addition to clinical data.

Based on the results of the association analysis between gene expression in patient iPS cell-derived motor neurons and estimated disease progression speeds, genes were sorted in ascending order of p-values. The expression levels of the top 5 or 10 genes, ranked by p-value, were used alongside available clinical information at the start of follow-up to predict estimated progression speeds. The mean squared error was compared between predictions using only clinical information (w/o) and those using both clinical information and gene expression levels (5 genes / 10 genes).
